# Mutation Pressure and Gene‐Specific Selection Shape Codon Usage Bias in the Chloroplast Genomes of *Isoetes*, an Ancient Aquatic Vascular Plant Lineage

**DOI:** 10.1002/ece3.74216

**Published:** 2026-09-17

**Authors:** Boxiong Li, Ying Xue, Dingqi Zhang, Guy Smagghe, Shixiong Liang, Shujing Wang, Yuye Ge, Zubaidai Aimaitijiang, Yunpeng Gai

**Affiliations:** ^1^ School of Grassland Science Beijing Forestry University Beijing China; ^2^ Department of Biology Vrije Universiteit Brussel (VUB) Brussels Belgium; ^3^ Institute of Entomology Guizhou University Guiyang China

**Keywords:** chloroplast genome, codon usage bias (CUB), evolutionary constraint, *Isoetes*, lycophytes, Phylogenomics, selection pressure, simple sequence repeats (SSRs), synonymous codon usage

## Abstract

*Isoetes* is one of the most ancient extant vascular plant genera and represents a distinct evolutionary lineage within lycophytes. Its chloroplast genome preserves molecular signatures relevant to early land plant evolution. However, the evolutionary forces shaping codon usage bias (CUB) in *Isoetes* chloroplast genomes remain poorly characterized. Here, we analyzed the chloroplast genomes of 60 *Isoetes* species to characterize CUB patterns and disentangle the contributions of mutation pressure and natural selection to chloroplast genome evolution in this basal vascular plant lineage. Chloroplast genome structure was highly conserved across species, with genome sizes of 142,880 bp to 145,535 bp and GC contents ranging from 37.56% to 38.18%. Parity rule 2 (PR2) plot analysis revealed a pronounced T/G bias at the third codon position, whereas neutrality plot analysis revealed no significant correlation between GC3 and GC12. All preferred codons (RSCU > 1) terminated in A or U, consistent with an AT‐ending preference. Simple sequence repeat (SSR) analysis revealed that SSRs are primarily distributed in intergenic regions, with mononucleotide repeats predominating, suggesting valuable molecular markers for phylogenetic studies. Our findings indicate that *Isoetes* chloroplast genomes exhibit weak but structured codon usage bias. Mutation pressure appears to be the dominant force establishing the AT‐rich compositional background, whereas natural selection acts in a gene‐specific manner, mainly through purifying selection and functional constraints on particular chloroplast genes. These results provide new insights into chloroplast genome evolution in basal vascular plants and highlight the combined effects of mutational bias and selective constraints on codon usage evolution. Unlike most previous chloroplast CUB studies focused on angiosperms, this genus‐wide analysis of *Isoetes* provides insight into codon usage evolution in a basal lycophyte lineage and suggests potential associations among CUB patterns, plastome conservation, functional constraints, and gene‐level evolutionary dynamics.

## Graphical abstract

This study analyzed the chloroplast genomes of 60 Isoetes species to characterize codon usage bias patterns and understand the evolutionary forces shaping this ancient lineage of aquatic vascular plants. The findings reveal a highly conserved chloroplast genome structure with relatively weak codon usage bias, primarily driven by mutation pressure toward an AT‐rich nucleotide composition. Despite this background mutational bias, purifying selection acts as the dominant evolutionary force preserving functional genes, while specific loci undergo targeted natural selection for environmental adaptation.

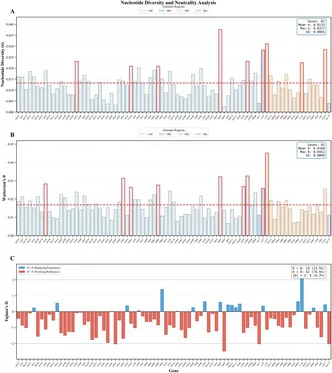

## 1 Background

The genus *Isoetes* (Isoetaceae, Isoetales) represents one of the most ancient extant lineages of vascular plants and the sole surviving genus of its family (Wikström et al. 2023). As a basal lycophyte lineage, *Isoetes* retains molecular and genomic signatures of key evolutionary transitions in land plant history, as reflected in its distinctive morphology, physiology, and life history traits (Raubeson and Jansen 1992). As a basal lycophyte lineage, *Isoetes* provides an informative system for investigating molecular features associated with early vascular plant evolution (Yang and Liu 2016). Despite its superficial morphological similarities to leek, *Isoetes* belongs to the lycophytes and reproduces via spores rather than seeds (Christenhusz and Chase 2014). Its life cycle and strict dependence on clean, oligotrophic aquatic habitats result in a highly specialized ecological niche, largely restricted to wetlands with low nutrient availability and minimal anthropogenic disturbance (Azzella et al. 2024).

These biological characteristics have made most *Isoetes* species highly vulnerable to environmental change. Many species face severe population declines worldwide and are threatened with extinction due to habitat destruction, eutrophication, and pollution (Deil 2005). In China, all known *Isoetes* species are classified as national first‐grade protected wild plants, reflecting both their rarity and their high scientific and ecological value. However, effective in situ conservation measures remain limited for most species, resulting in a pronounced mismatch between their conservation status and the urgency of their protection needs (Liu et al. 2005). The fossil record of *Isoetes* extends back to the Devonian period, approximately 360 million years ago (Wikström et al. 2023), predating the diversification of lycophytes and seed plants. As lycophytes (Cui et al. 2023; Karol et al. 2010), *Isoetes* species are often regarded as “living fossils”, providing a crucial evolutionary link between early terrestrial plants and modern vascular plant lineages.

In recent decades, research on *Isoetes* has expanded rapidly, encompassing studies of morphology (Troia et al. 2012; Liu et al. 2024), taxonomy (Troia and Greuter 2014; Troia and Greuter 2015; Bolpagni et al. 2021), ecology (Azzella et al. 2024), and, more recently, genomics (Cui et al. 2023). Concurrently, new species continue to be discovered, underscoring the hidden diversity within the genus (Fischer and Lobin 2022; Choi et al. 2008). A major milestone was achieved in 2023 with the completion of chromosome‐level sequencing of the tetraploid genome of *Isoetes sinensis* (Cui et al. 2023). This study revealed the loss of several key genes involved in environmental stress responses, providing a genomic explanation for the pronounced sensitivity of this species to habitat disturbance. These findings highlight the importance of understanding the molecular basis of environmental adaptation in *Isoetes*, particularly in the context of conservation under rapidly changing environmental conditions.

Although ex situ conservation programs for *Isoetes* have been initiated in China, the global conservation of the genus remains a complex and challenging task (Liu et al. 2005; Yang, Huang, et al. 2022; Troia et al. 2016). The worldwide distribution of *Isoetes* species is highly uneven, with many taxa confined to habitats characterized by strong and fluctuating environmental stresses, such as temporary pools subject to seasonal drought or high‐altitude wetlands with extremely low nutrient availability (Deil 2005). Several species are endemic to China, including *I. sinensis* , *Isoetes yunguiensis*, and *Isoetes yuhangensis* (Feng et al. 2019; Li et al. 2019). These taxa are critically endangered and afforded the highest level of legal protection. Notably, some *Isoetes* species have evolved unique physiological and biochemical strategies to cope with long‐term environmental stress. For example, *Isoetes howellii* has acquired crassulacean acid metabolism (CAM), a photosynthetic pathway that enhances water‐use efficiency (Richardson et al. 1984; Keeley and Busch 1984; Winter and Holtum 2014; Winter 2019). As one of the few lycophyte lineages known to possess CAM, *Isoetes* fixes carbon dioxide at night and releases it during the day, enabling survival in wetlands experiencing alternating flooding and drought. This distinctive metabolic adaptation underscores the evolutionary innovation of the genus and raises important questions regarding the genomic mechanisms underlying such physiological flexibility.

Despite the growth of genomic resources, the evolutionary dynamics of the *Isoetes* chloroplast genome remain poorly understood, particularly with respect to the determinants of codon usage bias (CUB). During its prolonged evolutionary history and repeated exposure to extreme or fluctuating environments, the *Isoetes* chloroplast genome retains a highly conserved overall architecture while showing localized sequence‐level variation (Raubeson and Jansen 1992; Cui et al. 2023). The coexistence of ancestral genomic features with derived adaptive traits makes *I. sinensis* and related species ideal models for investigating the relative contributions of natural selection and mutation pressure in shaping organelle genome evolution (Zhang et al. 2021; Yang et al. 2023). In particular, analysis of CUB in the chloroplast genome offers a promising approach for examining compositional bias, gene expression‐related constraints, and potential constraints on translational efficiency, thereby contributing to a molecular framework for understanding how early land plants adapt to terrestrial environments (Yang and Liu 2016).

Chloroplasts are the central organelles responsible for photosynthesis in green plants, algae, and other eukaryotic autotrophs, converting light energy into the chemical energy essential for life on Earth (Yin et al. 2017). According to endosymbiotic theory, chloroplasts originated from a cyanobacterial ancestor through an endosymbiotic event approximately 1–1.5 billion years ago (McFadden 2001; Chu et al. 2004). The chloroplast genome is typically a semiautonomous, circular DNA molecule with a conserved quadripartite structure consisting of a large single‐copy (LSC) region, a small single‐copy (SSC) region, and two inverted repeat (IR) regions. These regions encode genes essential for photosynthesis, transcription, translation, and ribosomal RNA (rRNA) biosynthesis. IRs contribute to genomic stability via homologous recombination, while conserved rRNA operons may influence codon usage patterns through transcriptional and translational regulation. Owing to their high expression levels, structural stability, and reduced risk of pollen‐mediated transgene escape, chloroplast genomes have also become important tools in plant biotechnology and evolutionary studies (Taberlet et al. 1991; Daniell et al. 2016).

CUB arises from the nonrandom use of synonymous codons and represents a fundamental mechanism influencing gene expression efficiency, accuracy, and regulation (Parvathy et al. 2022). From an evolutionary perspective, CUB reflects the combined effects of natural selection, mutation pressure, and genetic drift (Wang et al. 2018; Palidwor et al. 2010). Genomic features such as the GC content, gene length, recombination rate, and mutational biases further shape codon usage patterns through mutation–drift equilibrium (Wang and Hickey 2007). Investigating CUB in chloroplast genomes therefore provides insight into the balance between selective constraints on protein translation and neutral evolutionary processes. For ancient plant lineages such as *Isoetes*, such analyzes are particularly informative, as they shed light on long‐term evolutionary trajectories and the persistence of ancestral molecular features (Pereira, Giulietti, Pires, et al. 2021).

In recent years, plastome studies in ferns and lycophytes have shifted from single‐genome characterization toward comparative analyzes integrating genome structure, sequence divergence, RNA editing, codon usage, and selection pressure. For *Isoetes*, recent plastome‐based studies have greatly improved our understanding of phylogenetic relationships and plastome evolution within this ancient lineage. Chloroplast genomes of several key *Isoetes* species have been used to clarify evolutionary patterns in the genus (Pereira, Giulietti, Pires, et al. 2021), and plastome‐based phylogenomics has further helped resolve relationships among rare Neotropical *Isoetes* groups (Pereira, Giulietti, Prado, et al. 2021). Broader lycophyte plastome studies have also revealed substantial variation among *Isoëtaceae*, *Lycopodiaceae*, and *Selaginellaceae* in terms of plastome size, GC content, substitution rate, structural rearrangement, RNA editing, and gene loss (Chen et al. 2022). More recently, organellar‐genome analyzes of *Isoetes* highlighted exceptionally high frequencies of RNA editing, suggesting that post‐transcriptional modification may play an important role in maintaining organellar gene function in this ancient aquatic lineage (Pereira et al. 2024). Together, these studies indicate that lycophyte plastomes retain complex evolutionary signals that are highly relevant to early vascular plant evolution.

At the same time, codon usage bias has become an increasingly important perspective for understanding genome evolution and functional constraints in non‐seed vascular plants. A recent genome‐wide CUB analysis of representative lycophytes and ferns showed that several fern and lycophyte lineages exhibit distinct codon usage patterns, including differences in A/U‐ or C/G‐ending codon preferences, and that mutation pressure and natural selection jointly contribute to codon usage evolution (Xu et al. 2024). Similarly, recent chloroplast genome analyzes of Equisetum combined codon usage indices, ENC plots, PR2 plots, neutrality plots, and selection‐pressure analyzes to investigate the evolutionary forces shaping plastid genes (Sun et al. 2024). These studies demonstrate that CUB analysis can provide insights into translational constraints, nucleotide compositional bias, and adaptive or conservative evolutionary patterns in early‐diverging vascular plants.

However, despite these advances, genus‐scale analyzes of chloroplast CUB in *Isoetes* remain limited. Previous studies have mainly focused on plastome structure, phylogenomic relationships, RNA editing, or a small number of representative species, whereas the relative contributions of mutation pressure and natural selection to codon usage evolution across the genus have not been systematically evaluated. In particular, it remains unclear whether the AT‐rich background of *Isoetes* chloroplast genomes is primarily shaped by mutational bias, whether specific functional gene categories show stronger codon usage constraints, and how CUB patterns correspond to broader signals of plastome conservation and gene‐level selection. Addressing these questions is essential for understanding the molecular evolution of chloroplast genomes in basal vascular plants.

In this study, we used the chloroplast genomes of 60 *Isoetes* species to test three related hypotheses concerning codon usage evolution and plastome conservation in a basal lycophyte lineage. First, we hypothesized that the overall codon usage background of *Isoetes* chloroplast genomes is primarily shaped by AT‐biased mutation pressure, which should be reflected by low GC3s values, A/U‐ending preferred codons, high ENC values, and weak coupling between GC3 and GC12. Second, we hypothesized that selective or translational constraints act in a gene‐specific rather than genome‐wide manner, with stronger codon usage constraints expected in essential chloroplast functional modules such as photosystem and ribosomal protein genes. Third, we hypothesized that the highly conserved plastome architecture of *Isoetes* coexists with localized sequence variation, particularly in SSR loci and hypervariable genes, which may provide useful markers for phylogenetic and conservation studies.

To evaluate these hypotheses, we integrated codon usage indices, RSCU profiling, ENC‐GC3s deviation analysis, PR2 and neutrality plots, COA, SSR characterization, IR boundary comparison, phylogenetic and collinearity analyzes, Ka/Ks estimation, and nucleotide diversity analysis. Compared with previous CUB studies that have largely focused on angiosperms, this study targets *Isoetes*, a basal lycophyte lineage that diverged early in vascular plant evolution and retains distinctive aquatic or semiaquatic ecological features. The main contribution of this work is therefore not only to describe chloroplast CUB patterns, but also to provide a hypothesis‐driven framework for distinguishing mutation‐dominated compositional bias from gene‐specific functional constraints and localized plastome divergence in basal vascular plants.

## 2 Methods

### 2.1 Sequence Collection

The complete chloroplast genome sequences of *Isoetes* species were retrieved from the National Center for Biotechnology Information (NCBI) database. To ensure data quality and comparability, sequences were subjected to rigorous quality control procedures, including the removal of genomes with annotation inconsistencies, incomplete sequence information, or redundant taxonomic entries. After filtering, the chloroplast genome sequences from 60 *Isoetes* species were retained for subsequent analyzes (Table 1). We reannotated the sequences via GeSeq (Tillich et al. 2017) with reference annotations from related species, followed by manual curation. Start/stop codons and intron–exon boundaries were manually verified, and pseudogenes were distinguished from functional genes.

**TABLE 1:** GenBank accession numbers and sequence lengths of the *Isoetes* species used in this study.

Accession number	Species name	Sequence length
MH549640.1	*Isoetes malinverniana*	145,535
MZ596344.1	*Isoetes japonica*	145,517
ON193392.1	*Isoetes orientalis*	145,501
OM281829.1	*Isoetes yuhangensis*	145,501
PP213265.1	*Isoetes baodongii*	145,494
OM281828.1	*Isoetes changleensis*	145,494
ON060515.1	*Isoetes neoguineensis*	145,411
MK047605.1	*Isoetes yunguiensis*	145,355
ON060513.1	*Isoetes drummondii*	145,279
ON060512.1	*Isoetes pusilla*	145,224
MG792135.1	*Isoetes hyemalis*	145,133
MG668902.1	*Isoetes valida*	145,132
MG792143.1	*Isoetes virginica*	145,128
MG668893.1	*Isoetes flaccida var. flaccida*	145,126
MG792139.1	*Isoetes louisianensis*	145,119
MG792132.1	*Isoetes graniticola*	145,118
MG668896.1	*Isoetes melanopoda subsp. silvatica*	145,109
MZ603747.1	*Isoetes maritima*	145,097
MG599108.1	*Isoetes flaccida var. chapmanii*	145,096
MG668895.1	*Isoetes melanopoda subsp. melanopoda*	145,075
MG792141.1	*Isoetes mattaponica*	145,065
MG668898.1	*Isoetes mississippiensis*	145,061
MG668897.1	*Isoetes melanospora*	145,045
MH549641.1	*Isoetes piedmontana*	145,030
ON060510.1	*Isoetes echinospora* (*spiny‐sporedquillwort*)	144,998
MG668901.1	*Isoetes tegetiformans*	144,996
MZ603743.1	*Isoetes bolanderi*	144,991
MG668894.1	*Isoetes lithophila*	144,978
MZ603750.1	*Isoetes xherb‐wagneri*	144,975
ON060507.1	*Isoetes lechleri*	144,964
ON060514.1	*Isoetes sampathkumaranii*	144,947
ON060511.1	*Isoetes azorica*	144,936
MG668891.1	*Isoetes butleri*	144,912
ON060509.1	*Isoetes occidentalis*	144,912
ON060508.1	*Isoetes smithii*	144,865
MG668892.1	*Isoetes engelmannii* (*Engelmann'squillwort*)	144,817
MG668899.1	*Isoetes nuttallii*	144,680
ON060505.1	*Isoetes duriei*	144,291
ON060502.1	*Isoetes giessii*	143,699
ON060503.1	*Isoetes transvaalensis*	143,698
ON017796.1	*Isoetes aequinoctialis*	143,671
ON060504.1	*Isoetes biafrana*	143,629
ON060523.1	*Isoetes wormaldii*	143,603
MG019393.1	*Isoetes cangae*	143,380
MG019395.1	*Isoetes serracarajensis*	143,380
MW255542.1	*Isoetes anamariae*	143,364
MW255550.1	*Isoetes triangula*	143,357
MW255544.1	*Isoetes luetzelburgii*	143,346
MW255547.1	*Isoetes amazonica*	143,339
MW255549.1	*Isoetes panamensis*	143,331
MW255545.1	*Isoetes cipoensis*	143,330
MW255548.1	*Isoetes gardneriana*	143,326
MW255543.1	*Isoetes longifolia*	143,308
ON060521.1	*Isoetes australis*	143,237
ON060516.1	*Isoetes nigritiana*	143,221
ON060518.1	*Isoetes schweinfurthii*	143,220
ON060517.1	*Isoetes welwitschii*	143,215
ON060520.1	*Isoetes pallida*	143,173
ON060519.1	*Isoetes cubana*	143,143
MW255546.1	*Isoetes harleyi*	142,880

### 2.2 Codon Usage Indices

Protein‐coding sequences (CDSs) were extracted from the reannotated chloroplast genomes of 60 *Isoetes* species. Sequences with incomplete reading frames, internal stop codons, or ambiguous bases were excluded from codon usage calculations. Stop codons were not included in codon frequency analyzes. For each CDS, total GC content and codon‐position‐specific GC contents (GC1, GC2, and GC3) were calculated using custom scripts in Python v3.12.2. The effective number of codons (ENC) was calculated for each gene to quantify the overall degree of codon usage bias, and GC3s was used to represent GC content at synonymous third codon positions.

### 2.3 High‐Frequency Synonymous Codons

Relative synonymous codon usage (RSCU) values were calculated for protein‐coding genes using the Chloroplast Genome Analysis Pipeline (http://msgvd.genehub.com.cn/chloroplast2/). RSCU was calculated as the ratio between the observed frequency of a codon and the expected frequency under equal usage of synonymous codons encoding the same amino acid. Codons with RSCU > 1 were considered preferentially used, whereas codons with RSCU > 2 were regarded as strongly preferred. Methionine, tryptophan, and stop codons were excluded from synonymous codon preference comparisons because they lack synonymous alternatives or do not encode amino acids.

### 2.4 Repeat Sequence Identification

Simple sequence repeats (SSRs) are widely used as neutral molecular markers and provide insights into mutational dynamics and genome evolution. SSRs within the chloroplast genomes of the 60 *Isoetes* species were identified via the MISA‐web platform v2.1 (Beier et al. 2017). The MISA‐web parameters for identifying SSRs were set as follows: mononucleotides (10), dinucleotides (5), trinucleotides (4), tetranucleotides (3), pentanucleotides (3), hexanucleotides (3), heptanucleotides (3), octanucleotides (2), nonanucleotides (2), and decanucleotides (2). For each SSR locus, information on the genomic location, repeat motif type, repeat copy number, and functional context (protein‐coding region, intron, or intergenic spacer) was recorded. These data were subsequently used to evaluate patterns of repeat distribution and their potential association with mutational pressure in the *Isoetes* chloroplast genome.

### 2.5 Analysis of the IR Boundary Comparison

The IR regions of chloroplast genomes are among the most conserved structural elements; however, their boundary regions frequently undergo expansion and contraction. Such dynamics can alter gene copy number, lead to partial gene duplication, or result in pseudogenization at the IR‐LSC and IR‐SSC junctions, representing a common evolutionary mechanism underlying chloroplast genome length variation. To investigate IR boundary dynamics, we compared the junction regions of chloroplast genomes from Chinese endemic *Isoetes* species, globally distributed *Isoetes* taxa, and closely related *Selaginella* species. Visualization and comparative analyzes of IR boundaries were performed via IRscope (https://irscope.shinyapps.io/irapp/), enabling systematic examination of gene arrangement and boundary shifts among taxa.

### 2.6 ENC‐GC3s Plot Analysis

ENC‐GC3s plot analysis was used to evaluate whether codon usage patterns were consistent with mutational expectation based on GC3s composition. For each gene record, the expected ENC value under Wright's neutral expectation was calculated as: ENCexp = 2 + GC3s + 29/[GC3s^2^ + (1 − GC3s)^2^]. The deviation from the expected curve was calculated as ΔENC = ENCobs − ENCexp. Gene records with ΔENC < 0 were considered to be located below the expected curve, and records with ΔENC < −5 were defined as markedly deviating records. Functional categories of markedly deviating genes were assigned according to standard chloroplast gene annotations, including photosystem I, photosystem II, cytochrome b/f complex, ATP synthase, NADH dehydrogenase, RNA polymerase, ribosomal proteins, RuBisCO large subunit, YCF genes, and other protein‐coding genes. Scatter plots and summary statistics were generated in R v4.2.3 using RStudio and the ggplot2 package (Wright 1990).

### 2.7 Parity Rule 2 (PR2) Plot Analysis

PR2 analysis was performed to assess compositional asymmetry at synonymous third codon positions. For each gene, A3, T3, G3, and C3 were counted from synonymous codon positions after excluding stop codons and nondegenerate codons. The PR2 coordinates were calculated as A3/(A3 + T3) and G3/(G3 + C3). PR2 plots were generated in RStudio, with reference lines at 0.5 for both axes. Deviations from the central point were used to describe asymmetry in third‐position nucleotide usage.

### 2.8 Neutrality Plot Analysis

Neutrality plot analysis was conducted to evaluate the relationship between GC content at synonymous third codon positions and GC content at the first and second codon positions. For each gene, GC12 was calculated as the average of GC1 and GC2. Linear regression was performed using GC3 as the independent variable and GC12 as the dependent variable for each species. Regression slopes and coefficients of determination (R^2^) were recorded to evaluate the strength of association between GC3 and GC12. Scatter plots and regression analyzes were generated in RStudio.

### 2.9 Correspondence Analysis (COA)

COA was performed using RSCU values of chloroplast protein‐coding genes to summarize multivariate codon usage variation. The RSCU matrix was analyzed using the FactoMineR and factoextra packages in R. The first two COA axes were extracted and visualized, and the percentage of total variation explained by each axis was recorded. Because the first two axes explained only a limited proportion of total variation, COA was interpreted as an exploratory visualization of major codon usage trends rather than as definitive evidence of genome‐wide homogeneity.

### 2.10 Phylogenetic Tree Construction

To reconstruct a more robust phylogenetic framework for comparative plastome analyzes, a maximum‐likelihood phylogeny was inferred using IQ‐TREE based on concatenated chloroplast protein sequences. Complete chloroplast GenBank files from 70 lycophyte taxa were included, comprising 60 *Isoetes* taxa and 10 related outgroup taxa from Selaginella, Huperzia, and Lycopodium. Protein‐coding genes were extracted from the annotated GenBank files using custom Python scripts with Biopython. For genes with multiple copies within a plastome, the longest translated sequence was retained. Only protein‐coding genes shared by all 70 taxa were included to generate a fully occupied matrix without missing data. A total of 31 strictly shared chloroplast protein‐coding genes were retained for phylogenetic reconstruction. Amino acid sequences of each gene were aligned separately using MAFFT v7.505 with the “‐‐auto” strategy (Rozewicki et al. 2019; Katoh and Toh 2010; Katoh and Standley 2013; Katoh et al. 2019). Poorly aligned and ambiguous regions were removed from each single‐gene alignment using trimAl v1.5.rev1 with the “‐automated1” option (Capella‐Gutiérrez et al. 2009). The trimmed alignments were then concatenated into a chloroplast protein supermatrix using a custom Python script. The final concatenated matrix contained 70 taxa and 7023 amino acid sites. Maximum‐likelihood phylogenetic inference was performed using IQ‐TREE v2.0.7 (Minh et al. 2020). The best‐fitting amino acid substitution model was selected using ModelFinder according to the Bayesian information criterion. The selected model was JTT + F + I + G4. Branch support was assessed using 1000 ultrafast bootstrap replicates and 1000 SH‐aLRT replicates. In the resulting tree, node labels represent SH‐aLRT support values followed by ultrafast bootstrap support values. Nodes with SH‐aLRT ≥ 80 and UFBoot ≥ 95 were considered strongly supported. The final tree was rooted using Lycopodiales taxa, including Huperzia and Lycopodium, as outgroups. The phylogeny was used as a comparative framework for interpreting plastome collinearity, sequence divergence, and evolutionary patterns rather than for formal taxonomic revision.

### 2.11 Collinearity Synteny Analysis

Genome synteny analysis was conducted to identify conserved syntenic blocks and structural variations among *Isoetes* chloroplast genomes. By comparing gene order and orientation across species, this approach provides insight into the evolutionary history of structural rearrangements and facilitates the identification of conserved functional regions, as well as structural evidence supporting species differentiation and genome evolution. Collinearity visualization was performed via GenoPlotR v0.8.11 (Guy et al. 2010), which displays gene order conservation and structural variation by connecting homologous gene blocks across genomes. Genes were annotated and color‐coded according to functional categories, including photosystem genes (green), ribosomal protein genes (blue), RNA polymerase genes (orange), chlorophyll biosynthesis genes (yellow), and additional functional groups, as shown in the corresponding figures.

### 2.12 Ka/Ks Analysis

Orthologous chloroplast protein‐coding gene pairs were identified based on gene name matching. CDS alignments were generated using MAFFT v7.490 with the “‐‐auto” parameter and converted to AXT format. Ka and Ks values were calculated using KaKs_Calculator v2.0 under the model averaging method with the standard genetic code (Wang et al. 2010). During calculation, gaps and stop codons were removed according to the default behavior of KaKs_Calculator. Gene pairs with Ks = 0, undefined Ka/Ks values, premature stop codons, or unreliable alignments were excluded from downstream summary statistics. The Ka/Ks ratio was used as an indicator of evolutionary constraints: values greater than 1 were interpreted as elevated Ka/Ks ratios that may indicate candidate positive selection or relaxed selective constraints; values less than 1 reflect purifying selection, suggesting the removal of deleterious mutations; and values close to 1 are consistent with neutral evolution. This analysis provides insight into functional conservation and gene‐level evolutionary divergence among chloroplast genes. Genes were classified into functional categories on the basis of standard chloroplast gene nomenclature: photosystem I (*psaA*‐*psaJ*), photosystem II (*psbA*‐*psbZ*), cytochrome b/f complex (*petA*‐*petN*), ATP synthase (*atpA*‐*atpI*), NADH dehydrogenase (*ndhA*‐*ndhK*), RuBisCO large subunit (*rbcL*), RNA polymerase (*rpoA*‐*rpoC2*), large ribosomal proteins (*rpl2*‐*rpl36*), small ribosomal proteins (*rps2*‐*rps19*), hypothetical reading frames (*ycf* genes), and other protein‐coding genes (*matK*, *cemA*, *ccsA*, *infA*, *clpP*).

### 2.13 Similarity Analysis of Chloroplast Genomes

Comparative similarity analysis was performed to evaluate sequence conservation, evolutionary relationships, and divergence patterns among the chloroplast genomes. On the basis of the phylogenetic relationships inferred in this study, representative *Isoetes* species were selected for detailed comparison. The corresponding GenBank (gb) files were annotated via a custom Python script (https://github.com/Xwb7533/chloroplast_analysis). Annotated gb files were subsequently analyzed via mVISTA (Frazer et al. 2004; Dubchak et al. 2000) with the Shuffle‐LAGAN alignment algorithm (Brudno et al. 2003), enabling high‐resolution visualization of sequence similarity across entire chloroplast genomes. This approach facilitates the identification of conserved coding regions, divergent intergenic spacers, and lineage‐specific variation patterns.

### 2.14 Nucleotide Diversity Analysis

In this study, the complete chloroplast genome sequences of *Isoetes* species were utilized. Initially, multiple sequence alignments were performed for 81 shared protein‐coding genes (CDSs) across all the samples. To ensure analytical accuracy, manual adjustments were subsequently performed to eliminate obvious alignment errors. Nucleotide diversity (π), haplotype diversity, and Watterson's estimator (θ) were calculated via Python scripts. Additionally, Tajima's *D* values were computed to screen for deviations from neutral expectations. Finally, a sliding window analysis was conducted to characterize the distribution of nucleotide diversity values across the large single copy (LSC), small single copy (SSC), and inverted repeat (IR) regions.

## 3 Results

### 3.1 Characteristics of the *Isoetes* Chloroplast Genome

A comprehensive comparative analysis was performed on the chloroplast genomes of 60 *Isoetes* species, which represent a broad range of aquatic and semiaquatic taxa (Table S1). Despite substantial geographic and ecological diversity, the overall genomic architecture of *Isoetes* chloroplast genomes is highly conserved across species (Table 2).

**TABLE 2:** Detailed whole‐genome features, gene contents, and codon usage metrics of *Isoetes* chloroplast genomes.

GenBank accession number	Species Name	Total length (bp)	Total GC (%)	A%	T%	C%	G%	GC1%	GC2%	GC3%	GC3s	Coding region length (bp)	Coding region GC (%)	Noncoding region length (bp)	Noncoding region GC (%)	Protein‐coding gene num	rRNA gene num	tRNA gene num	Total gene num	Protein‐coding region GC (%)	rRNA GC (%)	tRNA GC (%)	Average RSCU	Average ENC
MG019393	Chloroplast *Isoetes cangae*	143,380	37.71	31.13	31.16	19.14	18.57	46.29	39.24	28.25	25.41	70,431	37.93	64,541	35.15	83	7	29	119	37.93	56.13	53.83	1	48.037
MG019395	Chloroplast *Isoetes serracarajensis*	143,380	37.71	31.13	31.16	19.14	18.57	46.31	39.21	28.23	25.39	70,452	37.92	64,520	35.16	83	7	29	119	37.92	56.13	53.83	1	48.015
MG599108	Chloroplast *Isoetes flaccida var. chapmanii*	145,096	37.99	30.98	31.02	19.25	18.74	46.48	39.41	28.48	25.64	71,260	38.13	62,011	34.48	84	8	37	129	38.13	55.88	54.59	1	48.133
MG668891	Chloroplast *Isoetes butleri*	144,912	38.02	30.98	31	19.27	18.75	46.5	39.44	28.48	25.63	71,251	38.14	61,836	34.53	84	8	37	129	38.14	55.86	54.59	1	47.906
MG668892	Chloroplast *Isoetes engelmannii* (Engelmann's quillwort)	144,817	38.05	30.96	30.99	19.29	18.76	46.54	39.49	28.48	25.64	71,215	38.17	61,778	34.55	84	8	37	129	38.17	55.88	54.64	1	48.111
MG668893	Chloroplast *Isoetes flaccida var. flaccida*	145,126	37.98	30.98	31.04	19.24	18.74	46.47	39.43	28.47	25.62	71,271	38.12	62,030	34.46	84	8	37	129	38.12	55.88	54.59	1	48.149
MG668894	Chloroplast *Isoetes lithophila*	144,978	38.02	30.98	31	19.26	18.76	46.47	39.39	28.5	25.65	71,255	38.12	61,898	34.51	84	8	37	129	38.12	55.88	54.59	1	48.075
MG668895	Chloroplast *Isoetes melanopoda* subsp. melanopoda	145,075	38	30.98	31.02	19.25	18.75	46.48	39.41	28.48	25.64	71,260	38.12	61,990	34.49	84	8	37	129	38.12	55.88	54.59	1	48.135
MG668896	Chloroplast *Isoetes melanopoda* subsp. silvatica	145,109	37.98	31.01	31.01	19.24	18.74	46.48	39.47	28.49	25.65	71,280	38.15	62,004	34.42	84	8	37	129	38.15	55.86	54.67	1	48.114
MG668897	Chloroplast *Isoetes melanospora*	145,045	38	30.99	31.01	19.26	18.74	46.5	39.49	28.45	25.62	71,235	38.15	61,985	34.47	84	8	37	129	38.15	55.88	54.59	1	48.102
MG668898	Chloroplast *Isoetes mississippiensis*	145,061	38.01	30.98	31.01	19.25	18.75	46.48	39.44	28.5	25.65	71,261	38.14	61,975	34.5	84	8	37	129	38.14	55.88	54.59	1	48.15
MG668899	Chloroplast *Isoetes nuttallii*	144,680	38.18	30.88	30.94	19.34	18.83	46.54	39.72	28.7	25.89	71,279	38.32	61,570	34.68	84	8	37	129	38.32	55.81	54.55	1	48.671
MG668901	Chloroplast *Isoetes tegetiformans*	144,996	38.02	30.98	31	19.26	18.76	46.5	39.49	28.48	25.63	71,260	38.15	61,911	34.51	84	8	37	129	38.15	55.83	54.67	1	48.136
MG668902	Chloroplast *Isoetes valida*	145,132	37.99	30.99	31.02	19.25	18.74	46.47	39.42	28.47	25.62	71,272	38.12	62,035	34.48	84	8	37	129	38.12	55.88	54.59	1	48.569
MG792132	Chloroplast *Isoetes graniticola*	145,118	37.99	31	31.01	19.26	18.74	46.52	39.49	28.49	25.64	71,226	38.17	62,067	34.45	84	8	37	129	38.17	55.83	54.67	1	48.13
MG792135	Chloroplast *Isoetes hyemalis*	145,133	38.04	30.98	30.98	19.27	18.77	46.51	39.49	28.5	25.66	71,260	38.16	62,048	34.38	84	8	37	129	38.16	55.86	54.67	1	48.124
MG792139	Chloroplast *Isoetes louisianensis*	145,119	38	31	31	19.25	18.75	46.5	39.48	28.49	25.65	71,260	38.15	62,034	34.39	84	8	37	129	38.15	55.86	54.67	1	48.115
MG792141	Chloroplast *Isoetes mattaponica*	145,065	37.98	31	31.01	19.25	18.74	46.49	39.47	28.49	25.65	71,260	38.15	61,980	34.44	84	8	37	129	38.15	55.86	54.67	1	48.101
MG792143	Chloroplast *Isoetes virginica*	145,128	38.04	30.96	31	19.28	18.76	46.46	39.41	28.47	25.62	71,275	38.11	62,023	34.37	84	8	37	129	38.11	55.88	54.42	1	48.329
MH549640	Plastid *Isoetes malinverniana*	145,535	38	30.98	31.02	19.25	18.75	46.57	39.62	28.49	25.7	71,185	38.23	62,428	34.39	84	8	38	130	38.23	55.86	54.74	1	48.216
MH549641	Plastid *Isoetes piedmontana*	145,030	37.99	31	31.02	19.25	18.73	46.37	39.38	28.41	25.57	69,680	38.05	63,511	34.64	83	8	37	128	38.05	55.85	54.59	1	48.031
MK047605	Chloroplast *Isoetes yunguiensis*	145,355	37.99	30.99	31.02	19.25	18.73	46.5	39.47	28.46	25.53	71,268	38.14	62,335	34.48	84	8	36	128	38.14	55.88	54.66	1	48.064
MW255542	Chloroplast *Isoetes anamariae*	143,364	37.75	31.1	31.15	19.17	18.58	46.48	39.12	28.12	25.29	70,146	37.91	61,462	34.17	82	8	36	126	37.91	55.83	54.38	1	47.556
MW255543	Chloroplast *Isoetes longifolia*	143,308	37.72	31.12	31.16	19.14	18.58	46.42	39.12	28.09	25.25	70,122	37.88	61,429	34.14	82	8	36	126	37.88	55.83	54.36	1	47.754
MW255544	Chloroplast *Isoetes luetzelburgii*	143,346	37.74	31.12	31.14	19.15	18.59	46.43	39.13	28.14	25.31	70,128	37.9	61,461	34.16	82	8	36	126	37.9	55.83	54.39	1	47.543
MW255545	Chloroplast *Isoetes cipoensis*	143,330	37.73	31.11	31.16	19.14	18.58	46.43	39.13	28.12	25.29	70,125	37.9	61,448	34.13	82	8	36	126	37.9	55.83	54.36	1	47.543
MW255546	Chloroplast *Isoetes harleyi*	142,880	37.76	31.09	31.15	19.16	18.6	46.44	39.11	28.13	25.3	70,134	37.89	60,989	34.19	82	8	36	126	37.89	55.83	54.36	1	47.547
MW255547	Chloroplast *Isoetes amazonica*	143,339	37.74	31.1	31.16	19.15	18.59	46.44	39.13	28.12	25.29	70,128	37.9	61,454	34.16	82	8	36	126	37.9	55.83	54.36	1	47.541
MW255548	Chloroplast *Isoetes gardneriana*	143,326	37.71	31.12	31.17	19.14	18.57	46.42	39.1	28.12	25.29	70,119	37.88	61,450	34.1	82	8	36	126	37.88	55.83	54.39	1	47.543
MW255549	Chloroplast *Isoetes panamensis*	143,331	37.73	31.12	31.16	19.14	18.58	46.4	39.11	28.13	25.29	70,128	37.88	61,446	34.15	82	8	36	126	37.88	55.83	54.39	1	47.567
MW255550	Chloroplast *Isoetes triangula*	143,357	37.73	31.1	31.16	19.15	18.59	46.43	39.11	28.11	25.27	70,152	37.88	61,448	34.17	82	8	36	126	37.88	55.83	54.36	1	47.538
MZ596344	Chloroplast *Isoetes japonica*	145,517	38	31.01	30.99	18.74	19.25	47.71	40.58	28.62	25.65	64,641	38.97	69,025	34.08	82	8	37	127	38.97	55.8	54.54	1	47.974
MZ603743	Plastid *Isoetes bolanderi*	144,991	38.01	30.97	31.02	19.25	18.75	46.48	39.43	28.45	25.6	71,220	38.12	61,954	34.52	84	8	37	129	38.12	55.87	54.56	1	48.107
MZ603747	Plastid *Isoetes maritima*	145,097	37.98	30.99	31.03	19.24	18.74	46.47	39.43	28.46	25.61	71,220	38.12	61,998	34.45	84	8	37	129	38.12	55.84	54.56	1	48.116
MZ603750	Plastid *Isoetes × herb‐wagneri*	144,975	38.01	30.97	31.02	19.25	18.75	46.47	39.43	28.45	25.6	71,220	38.12	61,922	34.52	84	8	37	129	38.12	55.88	54.56	1	48.116
OM281828	Chloroplast *Isoetes changleensis*	145,494	38	30.99	31.01	19.26	18.75	46.39	39.53	28.41	25.58	71,703	38.11	61,569	34.42	85	8	37	130	38.11	55.64	54.68	1	48.141
OM281829	Chloroplast *Isoetes yuhangensis*	145,501	38	30.99	31	19.26	18.75	46.41	39.49	28.37	25.53	71,246	38.09	62,445	34.58	84	8	37	129	38.09	55.9	54.68	1	48.068
ON017796	Plastid *Isoetes aequinoctialis*	143,671	38.1	30.92	30.99	19.29	18.81	47.84	41.31	29.49	26.56	39,533	39.55	92,287	35.24	61	8	37	106	39.55	55.78	54.44	1	46.839
ON060502	Plastid *Isoetes giessii*	143,699	38.1	30.91	30.99	19.29	18.81	47.84	41.32	29.5	26.57	39,536	39.55	92,312	35.24	61	8	37	106	39.55	55.75	54.52	1	46.86
ON060503	Plastid *Isoetes transvaalensis*	143,698	38.1	30.9	31	19.29	18.81	47.85	41.32	29.5	26.57	39,536	39.56	92,299	35.24	61	8	37	106	39.56	55.75	54.52	1	46.861
ON060504	Plastid *Isoetes biafrana*	143,629	38.11	30.91	30.98	19.29	18.82	47.84	41.31	29.49	26.56	39,533	39.55	92,245	35.26	61	8	37	106	39.55	55.78	54.44	1	46.839
ON060505	Plastid *Isoetes duriei*	144,291	37.94	31	31.06	19.23	18.71	47.86	40.95	29.29	26.36	40,547	39.36	91,900	35.05	63	8	37	108	39.36	55.79	54.43	1	47.141
ON060507	Plastid *Isoetes lechleri*	144,964	38.05	30.96	30.99	19.28	18.77	48.17	40.99	29.52	26.55	40,928	39.56	92,182	35.13	63	8	37	108	39.56	55.84	54.64	1	47.144
ON060508	Plastid *Isoetes smithii*	144,865	38.03	30.96	31.01	19.26	18.77	48.1	40.89	29.53	26.55	40,928	39.51	92,082	35.13	63	8	37	108	39.51	55.7	54.64	1	47.186
ON060509	Plastid *Isoetes occidentalis*	144,912	38.04	30.96	31	19.27	18.76	48.08	40.87	29.61	26.63	40,928	39.52	92,137	35.13	63	8	37	108	39.52	55.84	54.59	1	47.729
ON060510	Plastid *Isoetes echinospora* (spiny‐spored quillwort)	144,998	38.01	30.99	31	19.25	18.76	48.13	40.98	29.59	26.63	40,928	39.57	92,223	35.07	63	8	37	108	39.57	55.82	54.67	1	47.181
ON060511	Plastid *Isoetes azorica*	144,936	38.02	30.98	31	19.26	18.76	48.15	40.98	29.59	26.62	40,928	39.57	92,162	35.09	63	8	37	108	39.57	55.8	54.67	1	47.183
ON060512	Plastid *Isoetes pusilla*	145,224	38.07	30.97	30.96	19.28	18.78	48.04	41.24	29.61	26.7	38,807	39.63	94,569	35.24	61	8	37	106	39.63	55.84	54.65	1	47.278
ON060513	Plastid *Isoetes drummondii*	145,279	38.04	30.95	31.01	19.27	18.77	47.99	41.23	29.51	26.58	38,795	39.58	94,648	35.21	61	8	37	106	39.58	55.89	54.65	1	47.004
ON060514	Plastid *Isoetes sampathkumaranii*	144,947	38.07	30.94	30.99	19.3	18.78	48.04	41.24	29.56	26.65	38,810	39.61	94,289	35.25	61	8	37	106	39.61	55.75	54.65	1	47.273
ON060515	Plastid *Isoetes neoguineensis*	145,411	38.02	30.97	31.01	19.28	18.75	48.02	41.31	29.57	26.66	38,807	39.63	94,756	35.17	61	8	37	106	39.63	55.84	54.68	1	47.072
ON060516	Plastid *Isoetes nigritiana*	143,221	37.84	31.04	31.12	19.19	18.64	48.13	41.06	29.41	26.43	40,091	39.53	91,288	34.8	62	8	37	107	39.53	55.8	54.44	1	47.367
ON060517	Plastid *Isoetes welwitschii*	143,215	37.84	31.04	31.12	19.19	18.64	48.13	41.06	29.41	26.43	40,091	39.53	91,282	34.8	62	8	37	107	39.53	55.8	54.44	1	47.367
ON060518	Plastid *Isoetes schweinfurthii*	143,220	37.83	31.05	31.12	19.19	18.64	48.13	41.06	29.42	26.44	40,091	39.54	91,287	34.8	62	8	37	107	39.54	55.8	54.44	1	47.372
ON060519	Plastid *Isoetes cubana*	143,143	37.77	31.1	31.13	19.16	18.61	48.06	41.06	29.34	26.37	40,112	39.49	91,189	34.72	62	8	37	107	39.49	55.78	54.51	1	47.18
ON060520	Plastid *Isoetes pallida*	143,173	37.78	31.09	31.13	19.17	18.61	48.1	41.06	29.38	26.4	40,079	39.51	91,252	34.72	62	8	37	107	39.51	55.8	54.51	1	47.37
ON060521	Plastid *Isoetes australis*	143,237	37.56	31.18	31.26	19.05	18.51	48.19	40.56	28.88	25.8	40,365	39.21	91,008	34.49	64	8	37	109	39.21	55.75	54.51	1	46.878
ON060523	Plastid *Isoetes wormaldii*	143,603	38.09	30.92	30.98	19.33	18.76	47.62	41.56	29.46	26.39	34,958	39.54	96,885	35.48	53	8	36	97	39.54	55.67	54.08	1	45.964
ON193392	Chloroplast *Isoetes orientalis*	145,501	38	30.98	31.01	19.26	18.74	48.02	41.27	29.2	26.39	39,087	39.5	94,684	35.2	61	8	36	105	39.5	55.91	54.66	1	47.012
PP213265	Chloroplast *Isoetes baodongii*	145,494	38.01	30.99	31.01	19.26	18.75	46.51	39.5	28.39	25.51	71,052	38.13	62,638	34.55	83	8	37	128	38.13	55.91	54.68	1	48.316

Chloroplast genome sizes exhibited limited variation, ranging from 142,880 bp in *I. harleyi* to 145,535 bp in *I. malinverniana*, with a mean length of 144,462.62 ± 880.26 bp. This narrow size range is consistent with strong conservation of chloroplast genome organization within the genus. Consistent with typical plant plastomes, *Isoetes* chloroplast genomes presented an AT‐rich nucleotide composition. The mean GC content was 37.95% ± 0.14%, with minimal interspecific variation (from 37.56% in *I. australis* to 38.18% in *I. nuttallii* ). The low variance in GC content suggests highly conserved nucleotide composition across *Isoetes* species.

Analysis of the GC content at individual codon positions revealed pronounced positional asymmetry. The first codon position presented the highest GC content (GC1: 47.05% ± 0.76%), followed by the second position (GC2: 40.03% ± 0.87%), whereas the third codon position presented the lowest GC content (GC3: 28.77% ± 0.54%). The ENC ranged from 45.96 in *I. wormaldii* to 48.67 in *I. nuttallii* , with a mean value of 47.67 ± 0.54. These values, which approach the theoretical maximum of 61, indicate that overall CUB in *Isoetes* chloroplast genomes is relatively weak.

### 3.2 Cluster Analysis of RSCU Values

**FIGURE 1:**
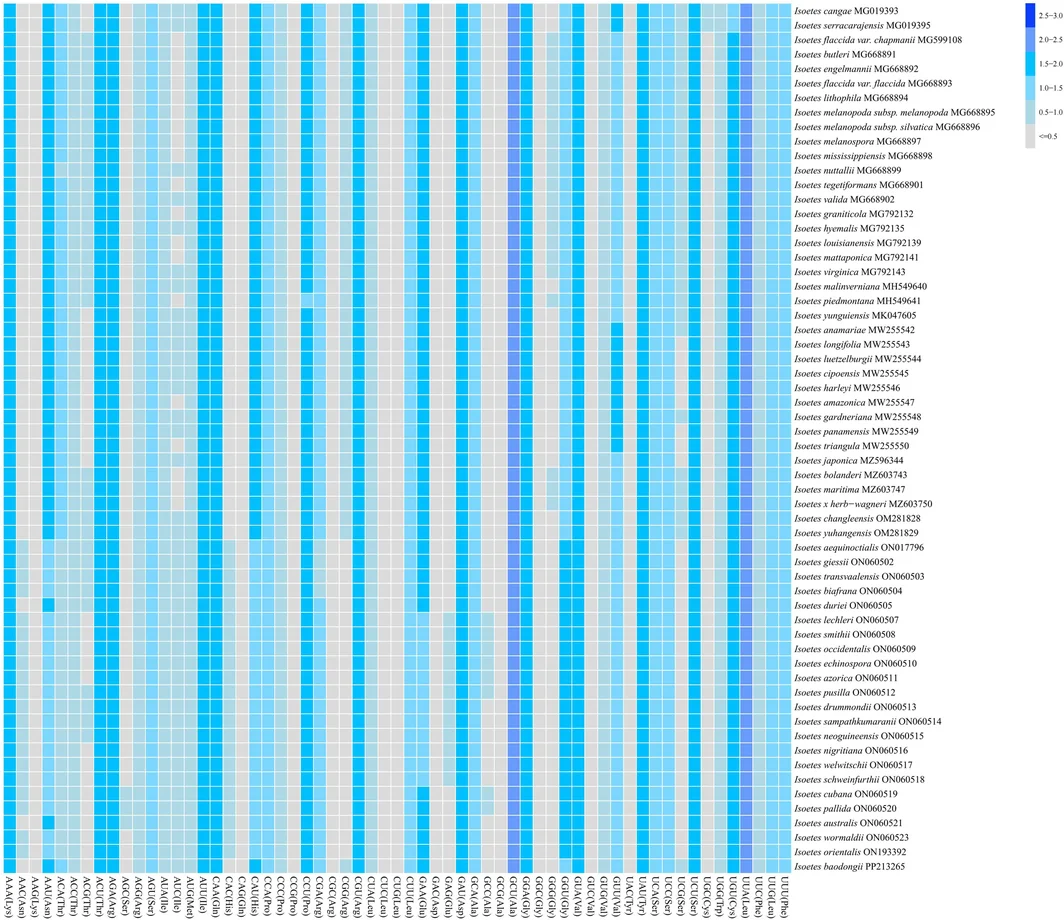
Heatmap of relative synonymous codon usage (RSCU) values across 60 *Isoetes* chloroplast genomes. Rows represent species; columns represent codons. Color intensity reflects RSCU magnitude (gray‐to‐blue gradient; scale: 0 to > 2). RSCU > 1 indicates overrepresented codons; RSCU > 2 indicates strongly preferred codons.

Cluster analysis based on RSCU values revealed consistent patterns of synonymous codon usage across the chloroplast genomes of the 60 *Isoetes* species (Figure 1). Codons encoding the same amino acids displayed unequal usage frequencies, indicating clear CUB. All codons with high RSCU values terminated with either A or U, which is consistent with the overall AT‐rich composition of the chloroplast genome and suggests a persistent mutational bias favoring A/T nucleotides.

Among these preferred codons, the leucine codon UUA was strongly enriched and terminated with A, in agreement with the general A/U‐ending preference. In contrast, the alanine codon GCU ends with U but contains G and C at the first two positions, suggesting that its preferential use may not be explained solely by third‐position A/U bias. This pattern suggests that additional factors, such as translational efficiency or functional constraints, may influence codon preference for certain amino acids.

The conservation of high‐RSCU codons across species implies that codon usage patterns are evolutionarily stable within *Isoetes*. Preference for specific synonymous codons may match the compositional bias of the genome. While A/U‐ending codon preference is common in plant chloroplast genomes, the particularly strong bias observed for leucine and alanine codons may reflect lineage‐specific codon usage features or functional constraints, although its relationship with the ecological niches occupied by *Isoetes* species remains to be tested.

### 3.3 Repeat Sequence Analysis

**FIGURE 2:**
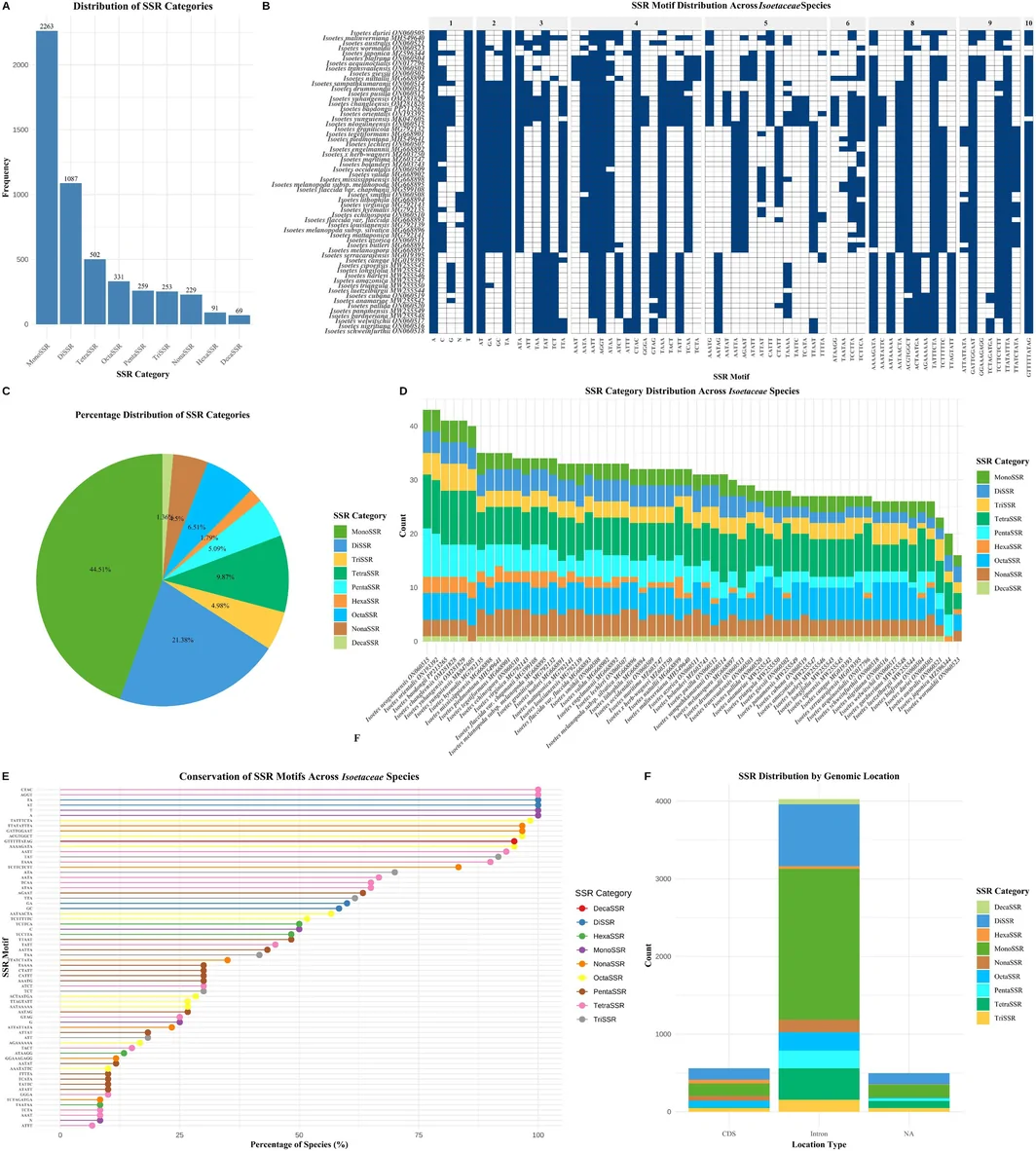
Simple sequence repeat (SSR) distribution in 60 *Isoetes* chloroplast genomes. (A) Frequency of SSR types. (B) Heatmap of SSR motif presence (blue) or absence (white); rows = species (clustered), columns = motifs (grouped by length). (C) Relative abundance of SSR types (pie chart). (D) SSR type composition per species (stacked bar chart). (E) SSR motif conservation; x‐axis = percentage of species containing the motif, dot color = SSR type, dot size = number of species. (F) SSR distribution by genomic region (CDS, intron, intergenic spacer). SSR types: MonoSSR = mononucleotide, DiSSR = dinucleotide, TriSSR = trinucleotide, TetraSSR = tetranucleotide, PentaSSR = pentanucleotide, HexaSSR = hexanucleotide, HeptaSSR = heptanucleotide, OctaSSR = octanucleotide, NonaSSR = nonanucleotide, DecaSSR = decanucleotide.

SSRs were systematically analyzed across the chloroplast genomes of *Isoetes* to characterize their distribution patterns, evolutionary features, and potential functional relevance (Table S2). A total of 5084 SSR loci were identified across the 60 *Isoetes* chloroplast genomes. Mononucleotide repeats were the most abundant SSR type, accounting for 2263 loci and 44.51% of all SSRs, followed by dinucleotide repeats with 1087 loci and 21.38% (Figure 2A,C). Together, mono‐ and dinucleotide repeats accounted for approximately two‐thirds of all detected SSRs, indicating that short repeat motifs dominate the SSR landscape of *Isoetes* plastomes. In contrast, longer SSR motifs, including tetranucleotide to decanucleotide repeats, were much less frequent, suggesting that repeat length expansion may be constrained in the chloroplast genome.

The predominance of short SSRs, especially A/T‐rich mononucleotide motifs, is consistent with the AT‐rich nucleotide composition of *Isoetes* chloroplast genomes. However, these SSR patterns provide information beyond simply confirming AT enrichment. The high abundance of short repeats is consistent with the possibility that replication slippage and local mutational instability contribute to microstructural variation in *Isoetes* plastomes. Because mononucleotide and dinucleotide repeats are particularly prone to length variation, these loci may represent mutational hotspots that accumulate variation faster than surrounding conserved regions.

SSR motif composition varied among species (Figure 2B,D). Heatmap and clustering analyzes revealed that several A/T‐rich motifs were widely shared across most species, indicating conserved repeat hotspots within the genus. At the same time, species‐specific or lineage‐biased motifs were detected in different *Isoetes* taxa. This coexistence of conserved and species‐specific SSR motifs suggests that the chloroplast genome of *Isoetes* maintains a stable overall repeat framework while allowing localized repeat variation. Such variation may provide useful markers for distinguishing closely related species, tracing lineage divergence, and assessing population‐level genetic diversity in conservation studies.

Analysis of SSR genomic distribution showed that most SSRs were located in noncoding regions, whereas fewer SSRs were found within protein‐coding sequences (Figure 2F). This uneven distribution suggests that SSR accumulation may be associated with both mutational bias and functional constraints. Noncoding regions can tolerate repeat expansion or contraction more readily, whereas SSRs within coding regions may be selectively constrained because length changes can disrupt reading frames or alter protein structure. Therefore, the preferential accumulation of SSRs in noncoding regions is consistent with the combined influence of replication slippage, AT‐biased mutation pressure, and stronger constraints against disruptive repeat variation in coding regions.

Overall, the SSR results indicate that repeat sequences contribute to chloroplast genome microvariation in *Isoetes*. Conserved SSR motifs may represent shared mutational hotspots across the genus, whereas species‐specific SSRs provide candidate molecular markers for species identification, phylogeographic analysis, and conservation genetics. Thus, SSR variation complements the codon usage and nucleotide diversity analyzes by identifying fine‐scale mutational features that may contribute to plastome divergence despite the high structural conservation of *Isoetes* chloroplast genomes.

### 3.4 Comparison of IR Boundary Regions

**FIGURE 3:**
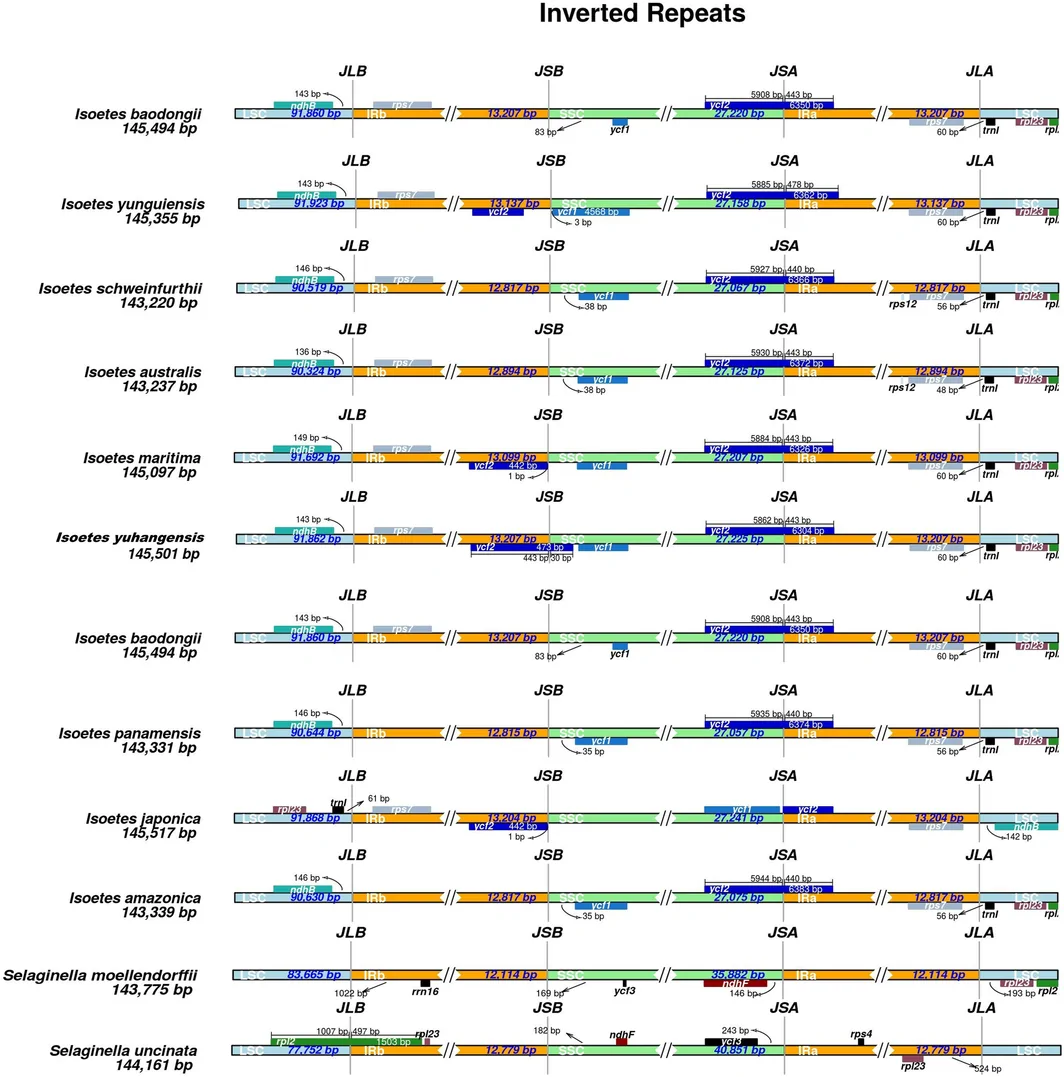
Inverted repeat (IR) boundary comparison among 10 *Isoetes* species and two Selaginella outgroups. Species names and genome sizes are shown. Colored bands indicate gene regions. Junctions between IRs and single‐copy regions are labeled: JLB (LSC/IRb), JSB (SSC/IRb), JSA (SSC/IRa), JLA (LSC/IRa). LSC = large single‐copy region; SSC = small single‐copy region; IR = inverted repeat.

Among extant lycophytes, *Selaginella* (Selaginellaceae) represents the closest extant relative of *Isoetes*. To evaluate structural variation in the chloroplast genomes, the IR boundary regions of 10 representative *Isoetes* species, including Chinese endemic taxa and globally distributed species, were compared with those of *Selaginella moellendorffii* and *Selaginella uncinata* (Figure 3).

The IR boundary architecture of *Isoetes* chloroplast genomes is highly conserved across species, with only minor expansion or contraction events observed. The pseudogenes *ycf1* and *ycf2* were consistently present, with *ycf2* typically spanning the SSC/IRa boundary. The LSC/IRa boundary commonly contains *rps7* and *trnI*, whereas the LSC/IRb boundary generally harbors *ndhB*. Notable deviations were observed in *I. yunguiensis*, *I. maritima* , *I. yuhangensis*, and *I. japonica* . In these species, the SSC/IRb boundary included a *ycf2* pseudogene; in *I. yuhangensis*, this pseudogene spanned the SSC/IRb junction, whereas in *I. japonica* , both the *ycf1* and *ycf2* pseudogenes were located at the SSC/IRa boundary without spanning it.

In contrast to the conservation observed in *Isoetes*, the two Selaginella species presented substantial and distinct IR boundary variations. Specifically, S. moellendorffii showed marked expansion at both the LSC/IRb and SSC/IRb boundaries, whereas *S. uncinata* was characterized by the rpl2 gene spanning the LSC/IRb boundary and a duplication of the rpl23 gene within the LSC/IRb region.

The consistent retention of *ycf1* and *ycf2* pseudogenes at the SSC/IRa boundary across *Isoetes* species, particularly the frequent spanning of *ycf2*, indicates highly conserved structural features within the genus, although the functional implications of this retention warrant further investigation. The contrasting IR boundary dynamics observed in *Selaginella* indicate fundamentally different evolutionary strategies of chloroplast genome organization between these closely related lycophyte lineages.

### 3.5 ENC‐GC3s Plot Analysis

**FIGURE 4:**
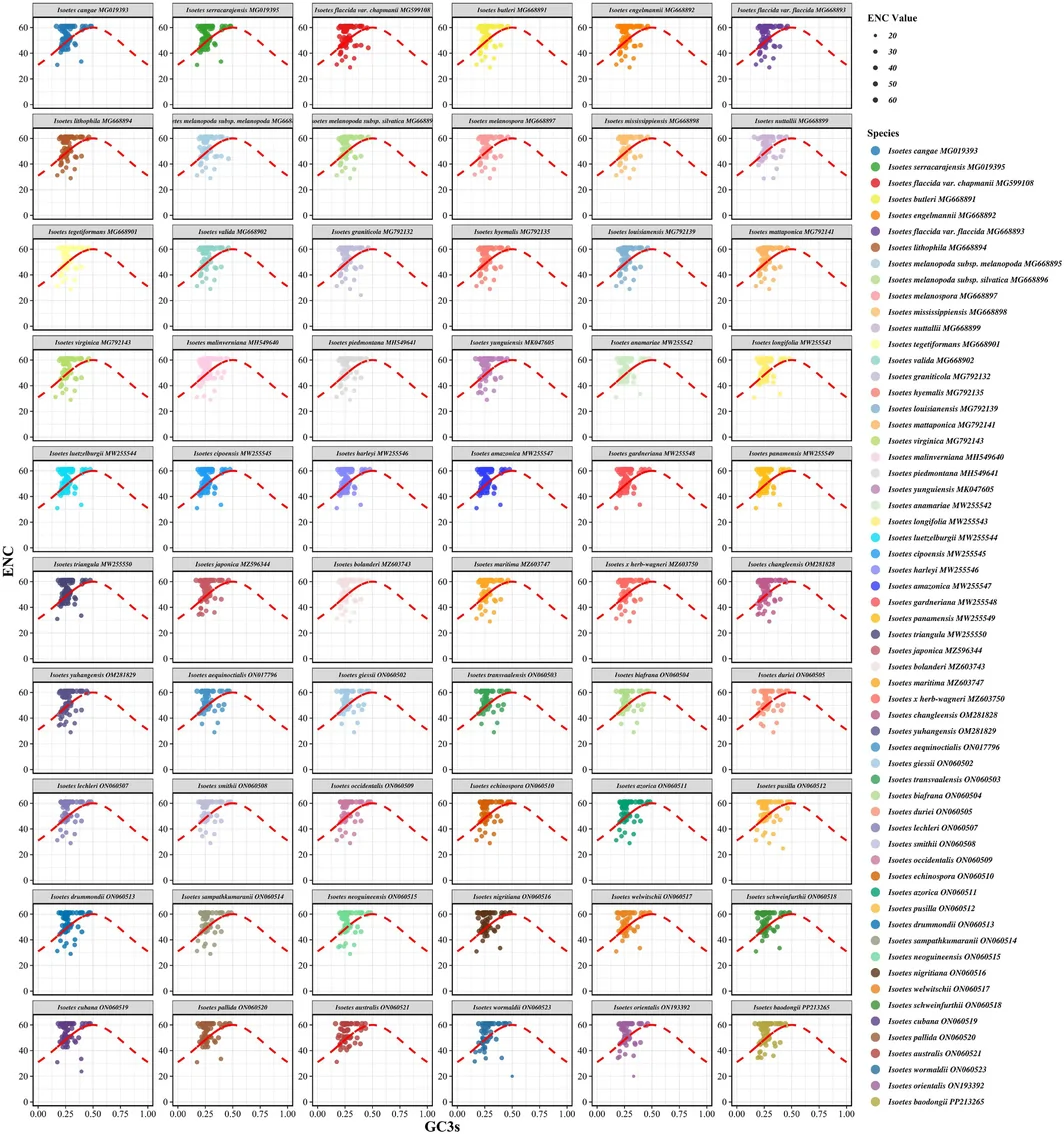
The correlation between GC3 and the effective number of codons (ENC) in the chloroplast genomes of 60 *Isoetes* species. Data points are mainly concentrated in the low‐GC3s and high‐ENC region, with GC3s values mostly between 0.2 and 0.4 and ENC values mainly between 45 and 60. The red dashed line represents Wright's expected ENC curve under mutational pressure alone, calculated as ENCexp = 2 + GC3s + 29/[GC3s^2^ + (1 − GC3s)^2^]. Individual species are represented through separate graphs, each identified by species name and accession number. The visualization employs a dark purple to yellow gradient. Each point corresponds to a chloroplast gene, with the point size and color intensity proportional to the ENC value; larger ENC values correlate with larger points and lighter colors.

ENC‐GC3s plot analysis was conducted to assess codon usage patterns and their evolutionary determinants in the chloroplast genomes of 60 *Isoetes* species (Figure 4). The ENC‐GC3s plots exhibited a highly conserved distribution pattern across all examined species. Most genes were concentrated in the high‐ENC and low‐GC3s region, with ENC values mainly ranging from 45 to 60 and GC3s values concentrated between 0.2 and 0.4. This distribution indicates generally weak codon usage bias and a strong AT preference at synonymous third codon positions.

To avoid relying solely on visual interpretation, we further quantified the deviation of each gene record from Wright's expected ENC curve (Table S3). The expected ENC value was calculated according to GC3s, and the deviation was estimated as ΔENC = ENCobs − ENCexp. Across 4523 analyzed gene records, 2787 records were located below the expected ENC curve, accounting for 61.62% of the total. Among them, 1036 records showed marked deviation from the curve, defined as ΔENC < −5, representing 22.91% of all records. These results indicate that although many genes follow the general expectation of AT‐biased mutation pressure, a substantial subset of genes displays stronger codon usage bias than expected from GC3s composition alone.

Functional classification further showed that the markedly deviating records were not randomly distributed among chloroplast gene categories. Photosystem II genes represented the largest proportion of markedly deviating records, accounting for 493 records and 47.59% of all marked deviations. Large ribosomal protein genes, photosystem I genes, and small ribosomal protein genes accounted for 165 records, 128 records, and 120 records, corresponding to 15.93%, 12.36%, and 11.58% of all marked deviations, respectively. In contrast, NADH dehydrogenase genes, RNA polymerase genes, and the RuBisCO large subunit gene showed no marked deviations under the ΔENC < −5 criterion.

These quantitative results indicate that most codon usage variation is consistent with the AT‐rich chloroplast genomic background, whereas marked deviations are concentrated in specific functional categories. In particular, the enrichment of markedly deviating records in photosystem II, photosystem I, and ribosomal protein genes suggests that codon usage in these essential chloroplast functional modules may be influenced by additional gene‐specific constraints, such as translational efficiency, expression demand, or purifying selection.

### 3.6 PR2 Plot Analysis

**FIGURE 5:**
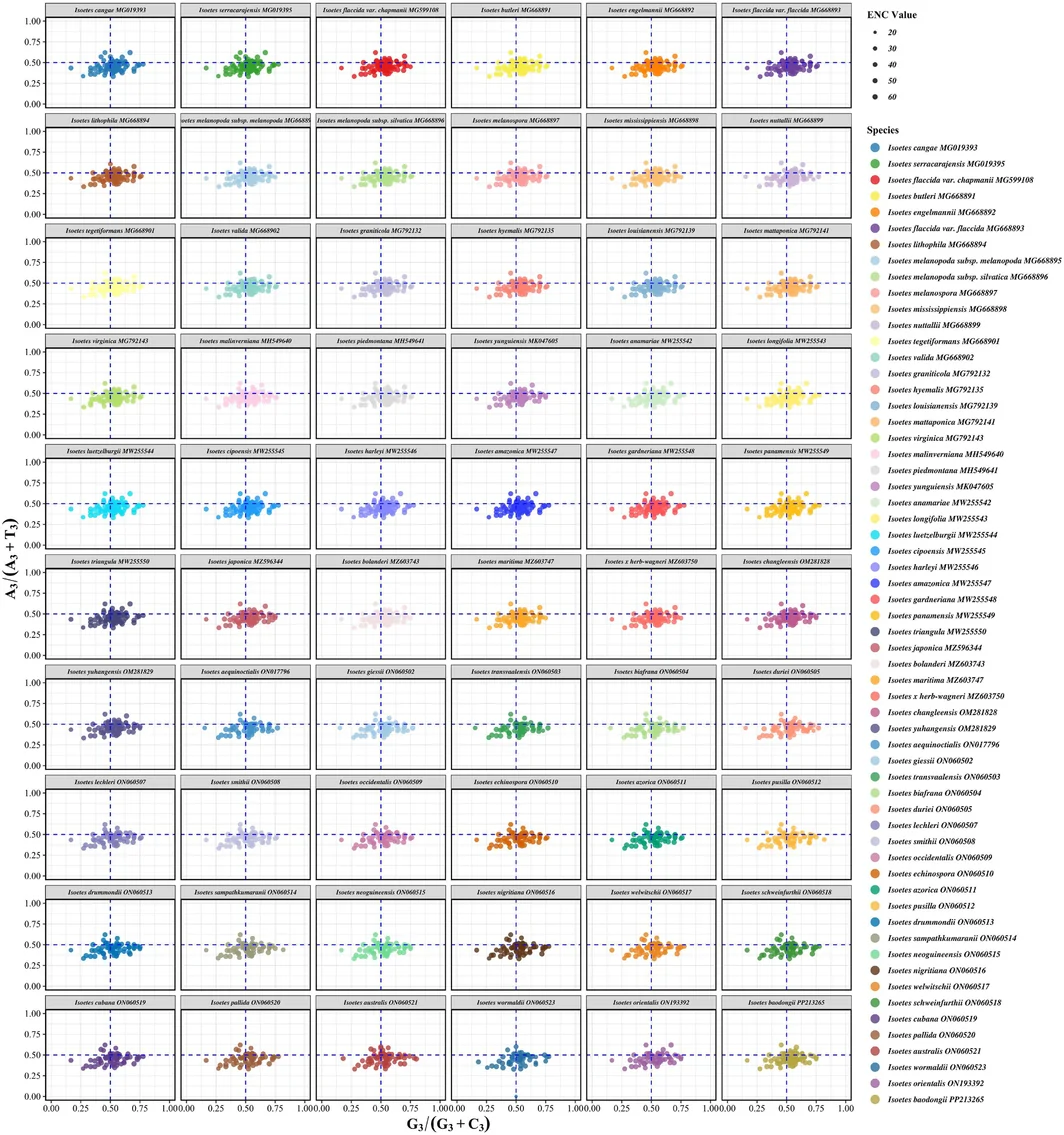
Relationships between A/T ratios and G/C ratios at the third position bases in chloroplast genes across 60 *Isoetes* species. Each data point represents an individual chloroplast gene, with point colors differentiating species and point sizes corresponding to ENC values, indicating base preferences at the third position. The horizontal and vertical lines intersect at 0.5, denoting the equilibrium positions for the GC and AT contents. The figure comprises multiple independent graphs for different species within the genus, each labeled with a species name and accession number to facilitate comparative analysis.

PR2 plot analysis was performed across the chloroplast genomes of 60 *Isoetes* species to investigate evolutionary drivers influencing nucleotide composition at the third codon position (Figure 5). Under a model of pure neutral evolution, nucleotide usage is expected to be balanced, with data points clustering around the center coordinate (0.5, 0.5). However, our results revealed a systematic and consistent deviation from this parity rule across all the examined species.

The majority of genes were predominantly clustered in the lower‐right quadrant of the plot. This distribution pattern corresponds to a coordinate range where G3/(G3 + C3) > 0.5 and A3/(A3 + T3) < 0.5, indicating a clear preference for pyrimidine T over purine A and purine G over pyrimidine C at the third codon position. Furthermore, the geometric shape of the gene distribution clouds provided insights into bias dynamics. The data points exhibited broader dispersion along the horizontal axis (G/C bias) than relatively tighter clustering along the vertical axis (A/T bias). This heterogeneity suggests that while the bias toward T over A is relatively stable and constrained across the genome, the bias toward G over C is more variable among different genes. The consistent deviation from the neutral origin (0.5, 0.5) across all 60 species suggests that codon usage patterns in *Isoetes* are unlikely to be governed solely by random drift and may also reflect strand‐specific mutational biases and selective constraints acting on the third codon position.

### 3.7 Neutrality Plot Analysis

**FIGURE 6:**
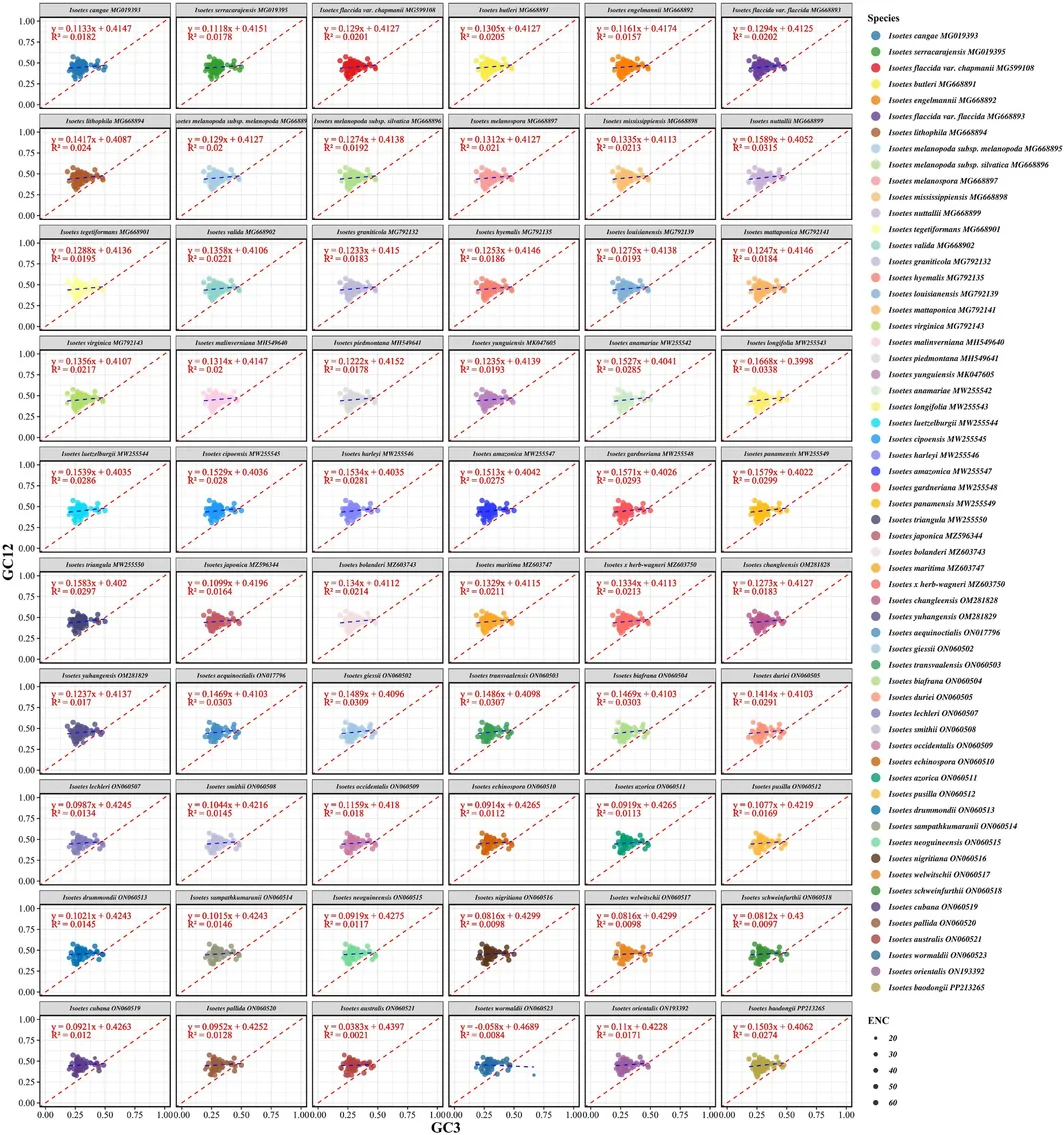
Neutrality analysis of the chloroplast genomes of 60 *Isoetes* species. Individual points represent chloroplast genes, with separate plots displaying different species within the genus, each identified by species name and accession number. The analysis included regression analysis of the GC content (GC3 and GC12) of the chloroplast genes, with the regression equation and R^2^ value indicated. The blue dashed line represents the regression line, whereas the red solid line indicates the Y = X relationship, indicating the correlation between GC3 and GC12.

Neutrality plot analysis was conducted for all 60 *Isoetes* species to quantify the relative contributions of mutation pressure and natural selection to codon usage patterns (Figure 6). This analysis plots the GC content of the first and second codon positions (GC12) against the third position (GC3). The plot reveals a distinct distribution pattern in which the vast majority of genes are clustered above the diagonal line. This finding indicates that the GC content at the first and second positions is significantly greater than that at the third position. The uneven distribution highlights a strong bias toward AT enrichment at the third codon position, whereas the GC content of the first two positions remains elevated, likely owing to functional constraints on amino acid composition.

Regression analysis further characterized the relationship between GC3 and GC12. The regression slopes (regression coefficients) across the 60 species were extremely low, ranging from −0.058 to 0.1668, with very weak coefficients of determination. In a neutrality plot, a slope of 1.0 implies complete neutral evolution, whereas a slope approaching 0 is generally interpreted as evidence that GC12 is relatively constrained and less coupled with GC3 variation. Our results show that the correlation between GC12 and GC3 is virtually nonexistent or weakly positive. Specifically, the low slopes suggest that while GC3 varies under mutational pressure, GC12 remains relatively conserved. This pattern indicates a decoupling between synonymous third‐position composition and the more functionally constrained first and second codon positions.

### 3.8 Correspondence Analysis (COA)

**FIGURE 7:**
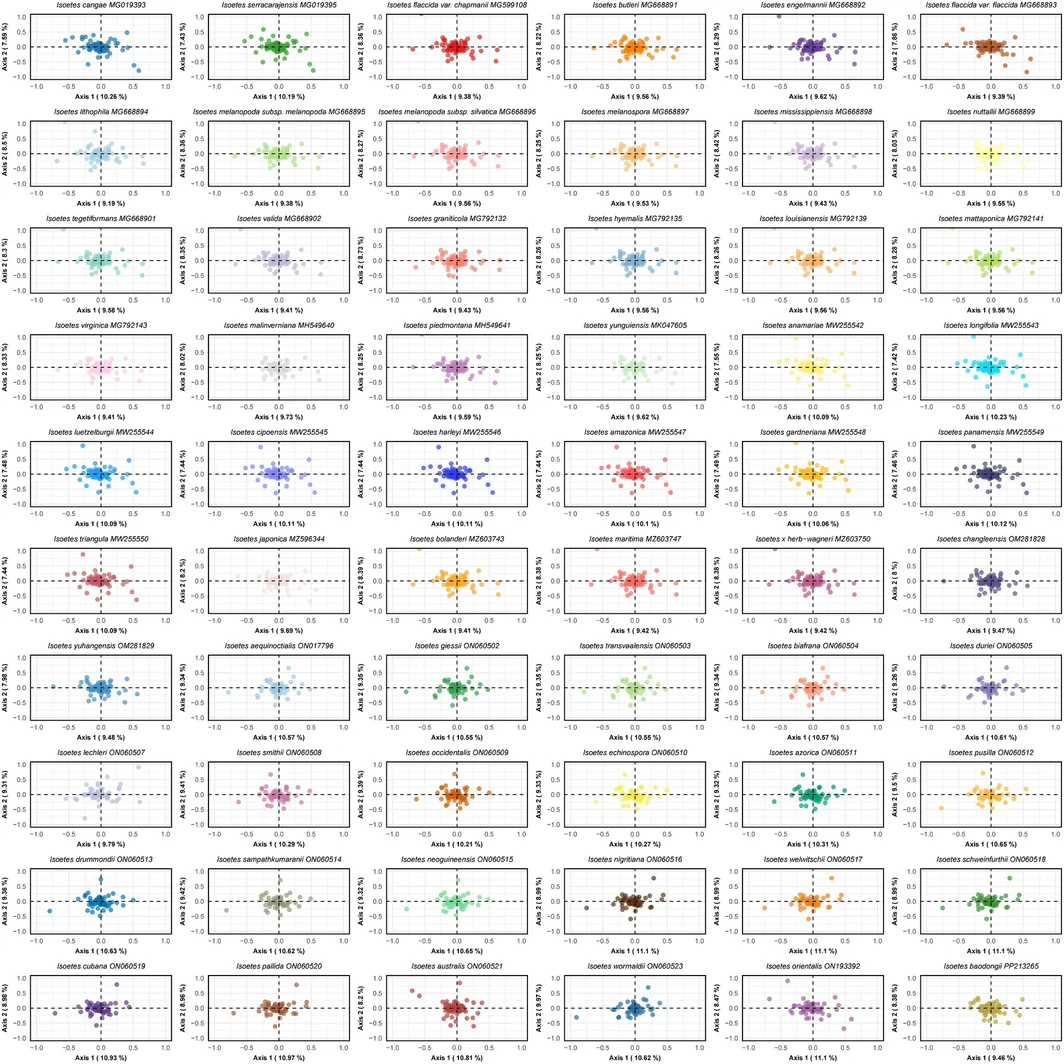
Correspondence analysis (COA) results for chloroplast genes in 60 *Isoetes* species. Individual points denote chloroplast genes, distinguished by species‐specific colors. The analysis uses Axis 1 and Axis 2 as coordinate axes, with marked contribution percentages for each axis. The dashed lines intersecting at zero points establish the center of the coordinate system. The spatial distribution of genes reflects major patterns of codon usage variation along the first two COA axes. Because the first two axes explain only a limited proportion of total variation, the plot should be interpreted as an exploratory visualization rather than definitive evidence of genome‐wide homogeneity.

Correspondence analysis was performed to visualize multivariate variation in codon usage among *Isoetes* chloroplast genes on the basis of RSCU values (Figure 7). Across the 60 species, the first two COA axes explained only a limited proportion of the total variation, with Axis 1 accounting for 9.71%–11.32% and Axis 2 accounting for 7.45%–9.64%. Thus, the two‐dimensional COA plots captured only part of the overall codon usage variation.

In the space defined by the first two axes, most genes were distributed near the origin and formed a relatively continuous cloud rather than distinct, isolated clusters. This pattern suggests that the major axes of codon usage variation are broadly similar among *Isoetes* chloroplast genes and that no strong differentiation was detected along the first two COA dimensions. However, because the variance explained by these two axes was relatively low, this result should be interpreted cautiously. The absence of clear separation in the two‐dimensional plots does not necessarily indicate complete genomic homogeneity, but rather suggests that codon usage variation is modest along the first two axes and may be distributed across additional dimensions.

Therefore, the COA results are consistent with the overall weak codon usage bias observed in the ENC analysis and the conserved A/U‐ending preference revealed by RSCU analysis. Nevertheless, they should be regarded as exploratory evidence for broadly similar codon usage trends rather than definitive evidence of genome‐wide homogeneity or uniform selective pressure across all chloroplast genes.

### 3.9 Phylogenetic and Genome Collinearity Analysis

**FIGURE 8:**
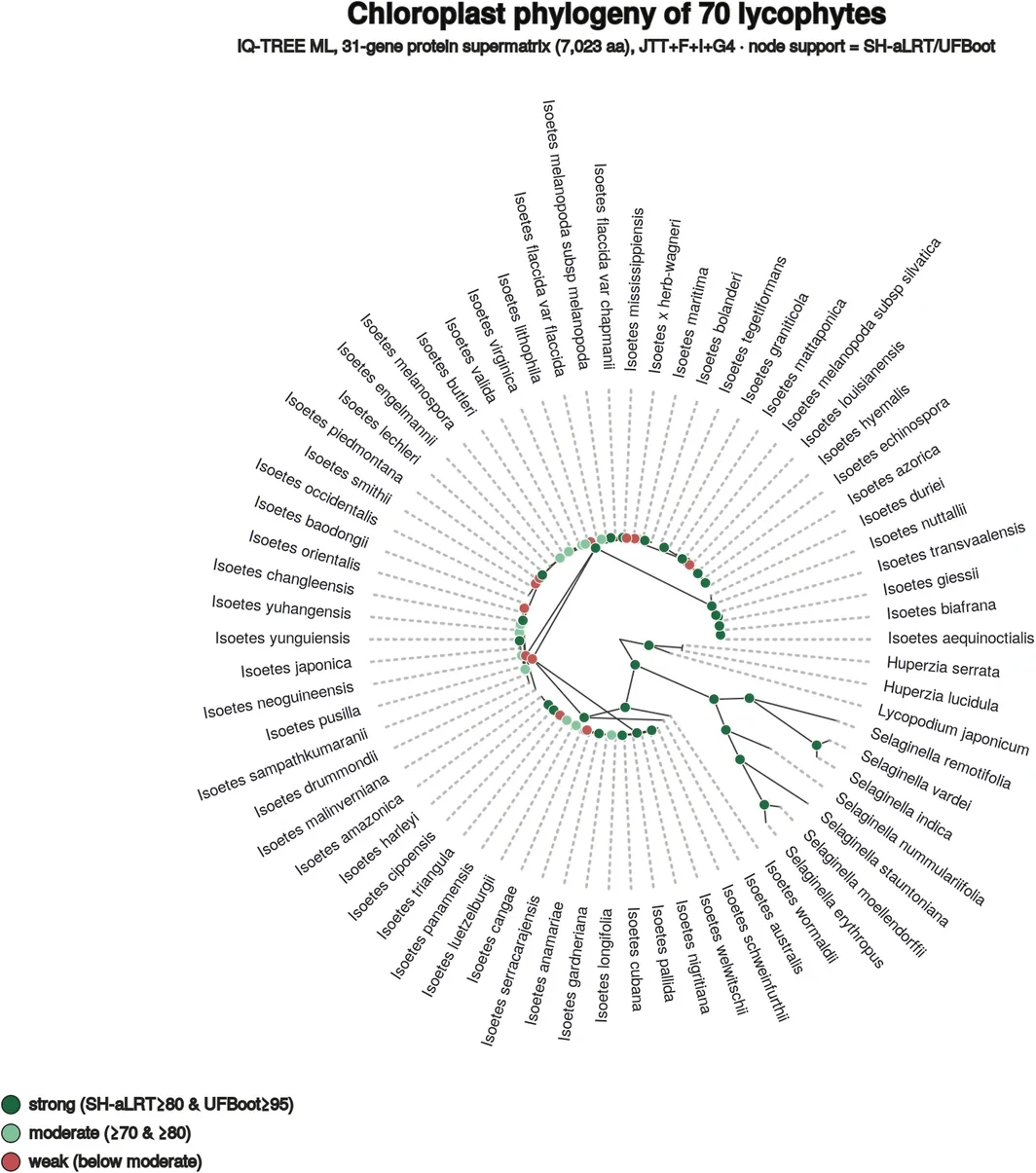
Maximum‐likelihood phylogenetic tree of 70 lycophyte taxa inferred from chloroplast protein sequences. The tree was constructed using IQ‐TREE based on a concatenated amino acid supermatrix of 31 shared chloroplast protein‐coding genes from 60 Isoetes taxa and 10 related outgroup taxa. The final alignment contained 7023 amino acid sites, and the best‐fitting substitution model selected by ModelFinder was JTT + F + I + G4. Node support was assessed using 1000 SH‐aLRT replicates and 1000 ultrafast bootstrap replicates, with support values represented as SH‐aLRT/UFBoot. Colored circles indicate node support categories: Strong support, SH‐aLRT ≥ 80 and UFBoot ≥ 95; moderate support, SH‐aLRT ≥ 70 and UFBoot ≥ 80; and weak support, below the moderate‐support threshold. The tree was rooted using Lycopodiales taxa as outgroups and was used as a comparative phylogenetic framework for representative taxon selection and plastome comparison.

To provide a robust phylogenetic framework for downstream comparative plastome analyzes, a maximum‐likelihood tree was reconstructed using IQ‐TREE based on a concatenated protein matrix of 31 shared chloroplast protein‐coding genes from 70 lycophyte taxa (Figure 8). The final alignment comprised 7023 amino acid sites, and ModelFinder selected JTT + F + I + G4 as the best‐fitting amino acid substitution model. Node support was evaluated using both SH‐aLRT and ultrafast bootstrap analyzes.

The resulting phylogeny clearly recovered the major lycophyte lineages included in this study. Lycopodiales, represented by Huperzia and Lycopodium, formed a distinct outgroup lineage. Selaginellales and Isoetales were each recovered as monophyletic groups, and Isoetales was placed as sister to Selaginellales. These deep relationships received strong support, indicating that the chloroplast protein dataset provided sufficient signal to resolve the major phylogenetic framework of the sampled lycophytes.

Within Isoetales, all sampled *Isoetes* taxa clustered together, supporting the monophyly of the genus in the present plastome‐scale analysis. However, relationships among many *Isoetes* species were characterized by short internal branches and variable support values. Several internal nodes received strong or moderate support, whereas weakly supported nodes were mainly concentrated within the Isoetes clade. This pattern suggests that chloroplast protein sequences are effective for resolving deep lycophyte relationships and broad intergeneric relationships, but provide limited resolution for some closely related *Isoetes* species at the amino acid level.

The relatively short branches and weak support values within parts of the *Isoetes* clade are consistent with the high conservation of chloroplast protein‐coding regions across the genus. This result supports the use of the IQ‐TREE phylogeny as a reliable comparative framework for selecting representative taxa and interpreting plastome collinearity, sequence divergence, and gene‐level evolutionary patterns. Nevertheless, the fine‐scale relationships among closely related *Isoetes* species should be interpreted cautiously, and future analyzes based on nucleotide‐level CDS alignments, noncoding plastome regions, or broader genomic datasets may provide additional resolution.

**FIGURE 9:**
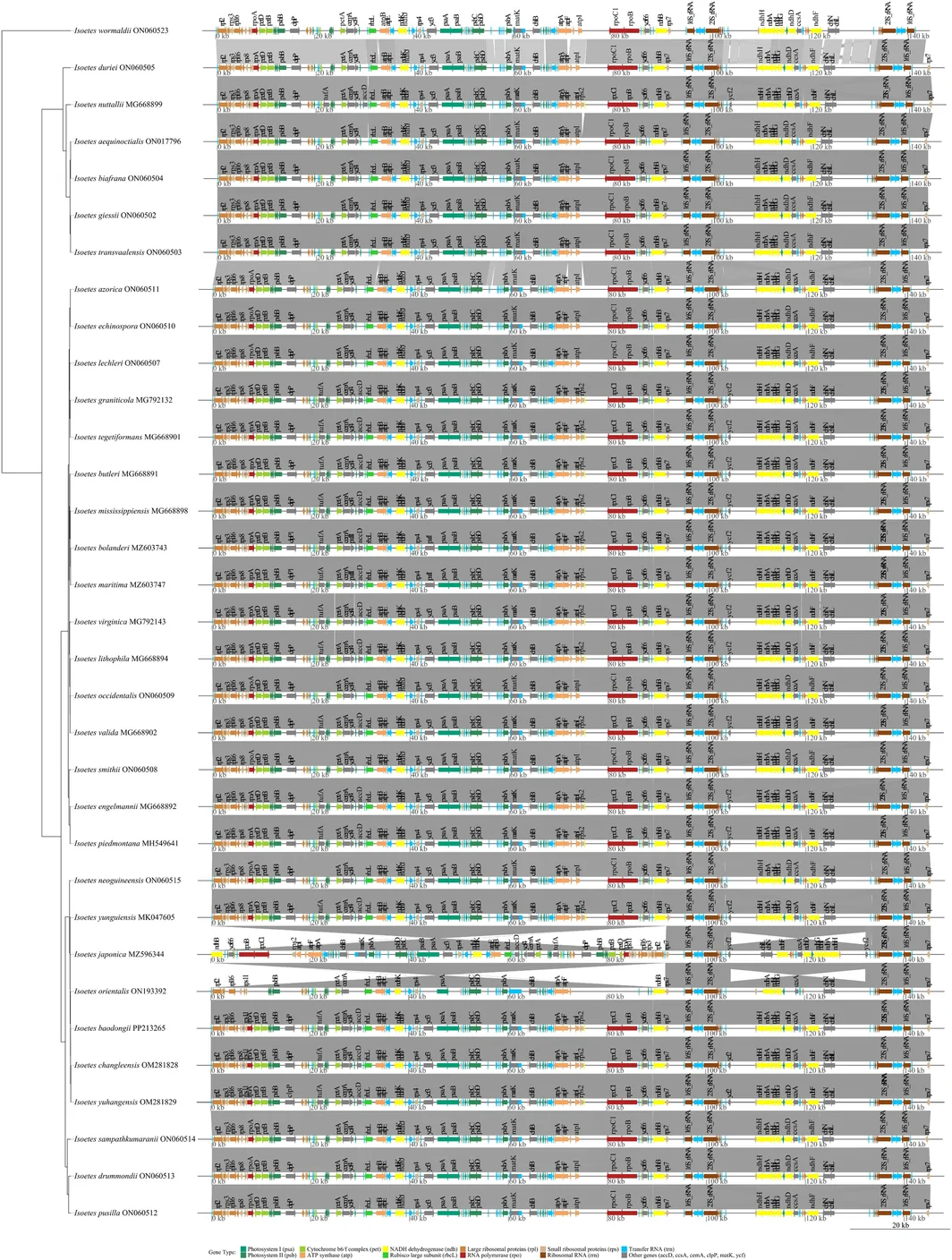
Genome synteny analysis of chloroplast genomes from 33 representative *Isoetes* species. Gray blocks connecting genomes represent conserved collinear regions. Gene annotations are color‐coded by functional category: Photosystem (green), ribosomal proteins (blue), RNA polymerase (orange), and other functions (as indicated in the legend).

On the basis of this phylogenetic framework, 33 representative *Isoetes* species spanning major clades, including both widely distributed and geographically restricted taxa, were selected for genome collinearity analysis. Comparative analysis of their chloroplast genomes demonstrated a high degree of structural conservation (Figure 9). Gene order, orientation, and functional organization were largely consistent across species, particularly for core photosynthesis‐related gene clusters.

The extensive collinearity observed among *Isoetes* chloroplast genomes indicates that plastome architecture is highly conserved across the sampled taxa. This structural conservation provides independent support for the overall stability of *Isoetes* chloroplast genomes and suggests that the main comparative conclusions of this study do not rely solely on the exact topology of the IQ‐TREE phylogeny.

### 3.10 Patterns of Selection Pressure Across Chloroplast Genes

**FIGURE 10:**
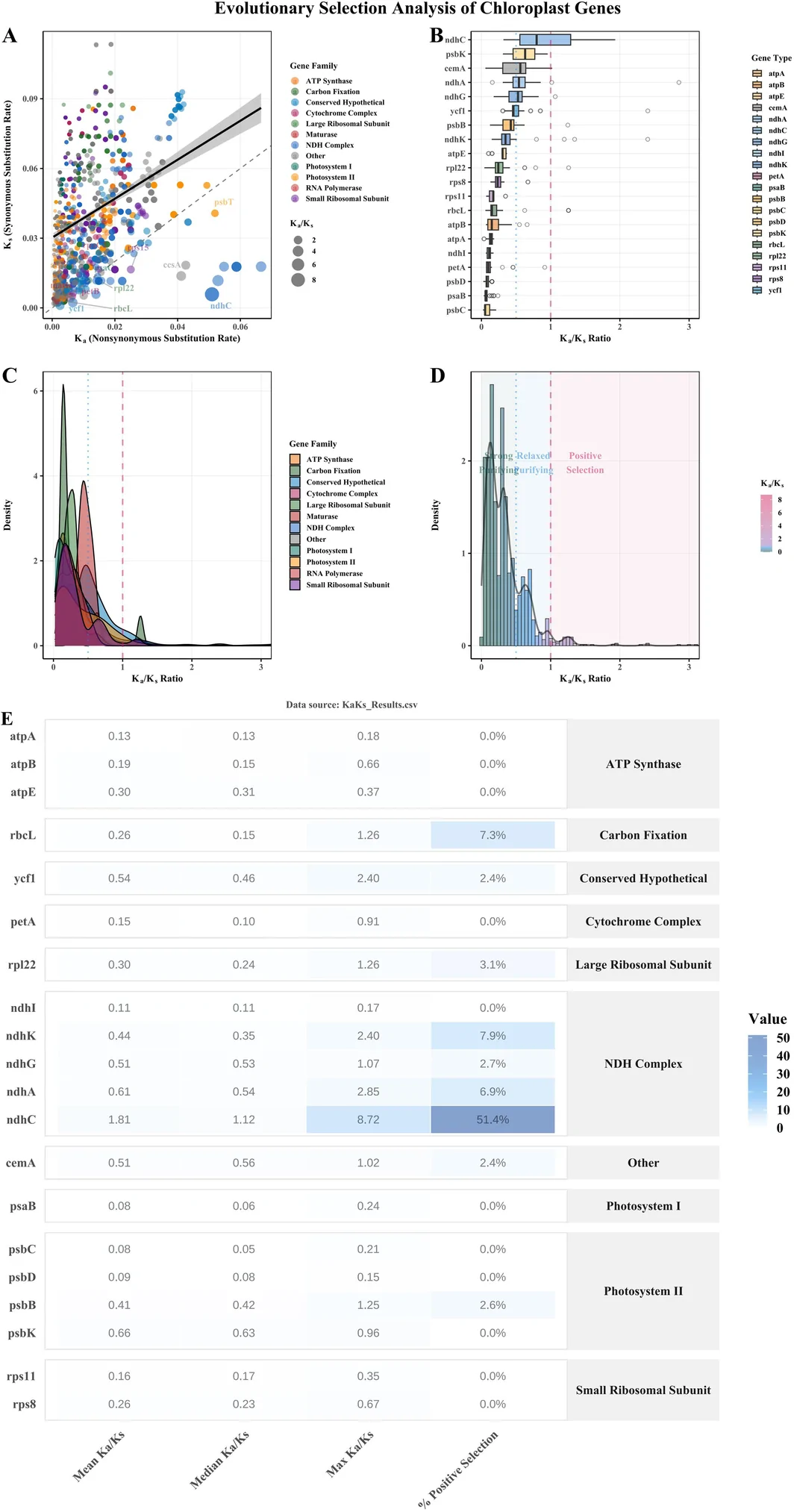
(A) Scatter plot of nonsynonymous substitution rate (Ka) versus synonymous substitution rate (Ks). Each point represents a homologous gene comparison; colors indicate gene families, and point size indicates the Ka/Ks ratio. The dashed line indicates Ka = Ks. (B) Boxplot of Ka/Ks ratios for representative chloroplast genes, arranged by median Ka/Ks value. Dashed reference lines indicate Ka/Ks = 0.5 and Ka/Ks = 1. (C) Density distribution of Ka/Ks ratios among major chloroplast gene families. (D) Overall distribution of Ka/Ks ratios across all analyzed gene comparisons. Background regions indicate Ka/Ks < 0.5, 0.5 ≤ Ka/Ks < 1, and Ka/Ks ≥ 1. (E) Summary of genes with relatively elevated Ka/Ks values, including mean, median, maximum Ka/Ks, and the proportion of comparisons with Ka/Ks > 1.

To investigate the evolutionary forces acting on *Isoetes* chloroplast genes, we conducted a comparative analysis of nonsynonymous substitution rates (Ka), synonymous substitution rates (Ks), and Ka/Ks ratios (Table S4). The Ka/Ks scatter plot showed that synonymous substitutions generally exceeded nonsynonymous substitutions for most genes (Figure 10A). Most data points were distributed below the neutral expectation line of Ka = Ks, indicating that purifying selection is the predominant evolutionary force maintaining the functional integrity of the chloroplast genome.

Analysis of individual genes revealed substantial heterogeneity in selective constraints among chloroplast genes (Figure 10B,E). Photosystem I and II genes, including *psaB*, *psbC*, and *psbD*, exhibited very low mean Ka/Ks ratios, generally ranging from 0.08 to 0.09, and showed no cases of Ka/Ks > 1. These results indicate strong functional conservation of the core photosynthetic machinery. The *rbcL* gene, which encodes the large subunit of RuBisCO, also showed strong conservation, with a median Ka/Ks value of 0.15, although a small proportion of comparisons showed elevated values.

In contrast, several NDH complex genes displayed relatively elevated Ka/Ks ratios. Among them, ndhC was the most prominent outlier, with a mean Ka/Ks ratio of 1.81, a median value of 1.12, and a maximum value of 8.72. In addition, 51.4% of the examined comparisons for ndhC showed Ka/Ks values greater than 1. Other NDH genes, such as ndhK and ndhA, also showed elevated maximum Ka/Ks values of 2.40 and 2.85, respectively. These results suggest that the NDH complex represents a relatively dynamic region of the Isoetes chloroplast genome.

However, elevated Ka/Ks ratios should be interpreted cautiously. Although Ka/Ks values greater than 1 may indicate candidate signals of positive selection, they may also reflect relaxed selective constraints, lineage‐specific rate acceleration, reduced functional constraint, or stochastic effects associated with low synonymous substitution rates. Therefore, the elevated Ka/Ks ratios observed in ndhC and some other NDH genes should be regarded as evidence of relaxed or altered selective constraints rather than definitive proof of adaptive positive selection.

Density analysis at the gene family level further supported this interpretation (Figure 10C,D). Photosystem genes and RNA polymerase genes showed narrow density peaks concentrated in the strong purifying selection region, whereas the NDH complex exhibited a broader distribution with a tail extending into the Ka/Ks > 1 region. Overall, these results indicate that the *Isoetes* chloroplast genome is primarily shaped by purifying selection, while a limited number of genes, especially those in the NDH complex, show elevated evolutionary rates that may reflect relaxed selection, lineage‐specific rate variation, or possible functional divergence.

### 3.11 Comparative Genome Similarity Analysis

**FIGURE 11:**
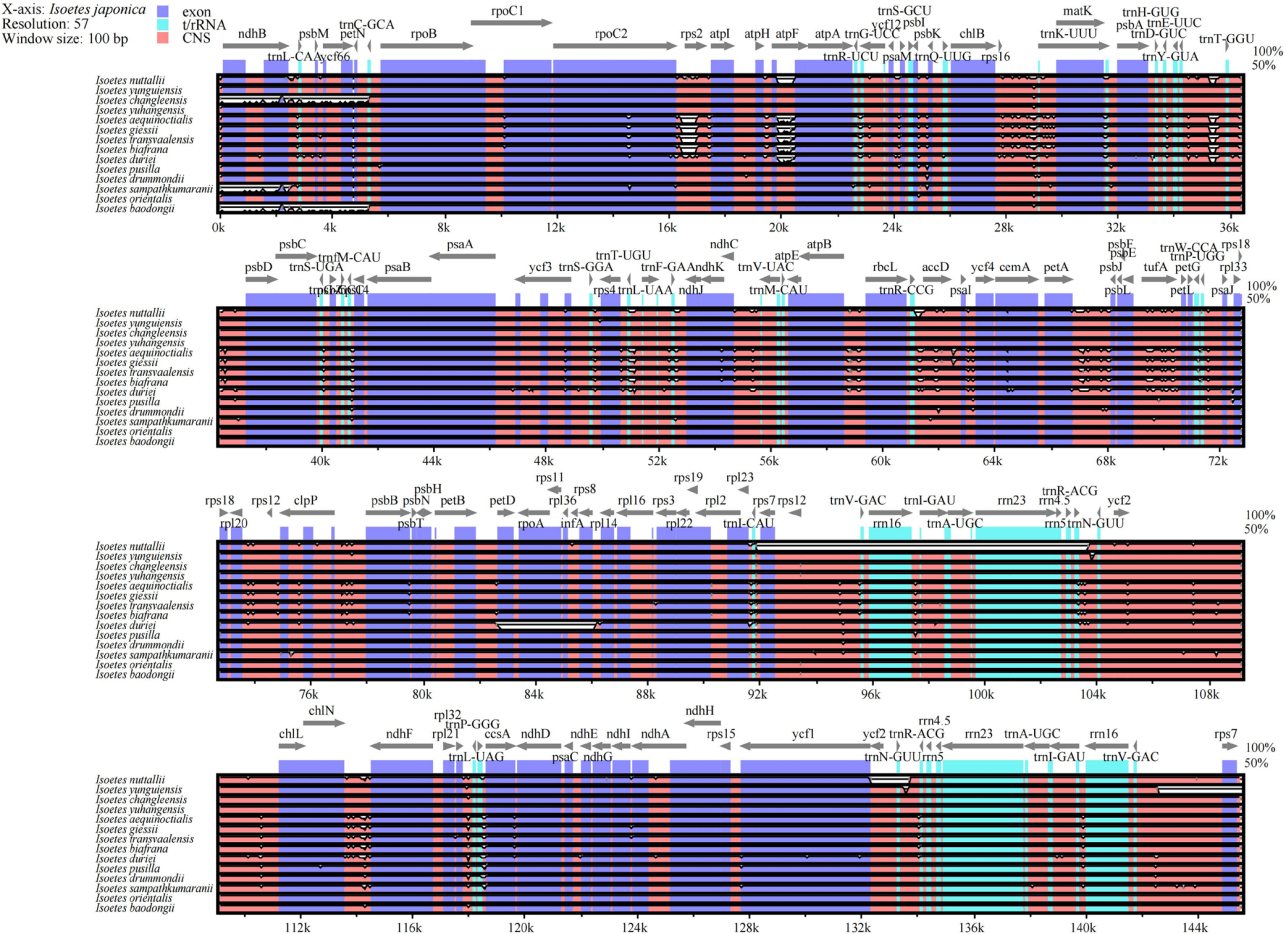
Visualization of the genome alignment of the chloroplast genomes of 15 *Isoetes* species. Using *Isoetes japonica* as the reference genome, this analysis was conducted via mVISTA. The visualization includes annotations of gene names and coding regions, with genome coordinates displayed on the x‐axis and species distributions on the y‐axis. The alignment similarity between regions is represented by horizontal bars, with percentages ranging from 50% to 100%.

On the basis of the phylogenetic tree, 15 representative *Isoetes* species, including several Chinese endemic species and members of minor branches, were selected for a detailed comparative genomic analysis. The chloroplast genome of *Isoetes japonica* was employed as a reference for determining sequence divergence via the mVISTA program (Figure 11). The global alignment results revealed a high degree of sequence similarity and collinearity across the *Isoetes* genus, confirming the conservation of the genomic structure. The majority of sequence divergence was concentrated in noncoding regions (CNS), particularly in intergenic spacers (IGS) and introns, which appeared as distinct “valleys” of low similarity (< 50%) in the mVISTA plot. In contrast, the RNA‐coding regions and most protein‐coding genes maintained high conservation, with identity levels generally exceeding 90%.

However, substantial variation was detected in specific coding regions. Notably, the genes *ndhB*, *rps2*, and *atpF* showed relatively elevated divergence among the annotated genes. The pronounced divergence in *rps2* may reflect the relaxed selective constraints or potential pseudogenization events often observed in this gene within lycophytes, whereas the variation in *atpF* and *ndhB* is likely driven by the higher mutation rates within their intronic sequences. At the species level, the chloroplast genomes of *Isoetes nuttallii* , *Isoetes yunguiensis*, and *Isoetes duriei* presented the most substantial sequence variation compared with the reference *I. japonica* . These species display widespread regions of reduced similarity across both coding and noncoding regions, which is consistent with their greater phylogenetic distance. Conversely, species such as *I. baodongii* and *I. orientalis* presented highly conserved profiles with minimal deviations from the *I. japonica* genome, supporting their close evolutionary relationships.

### 3.12 Nucleotide Diversity Analysis

**FIGURE 12:**
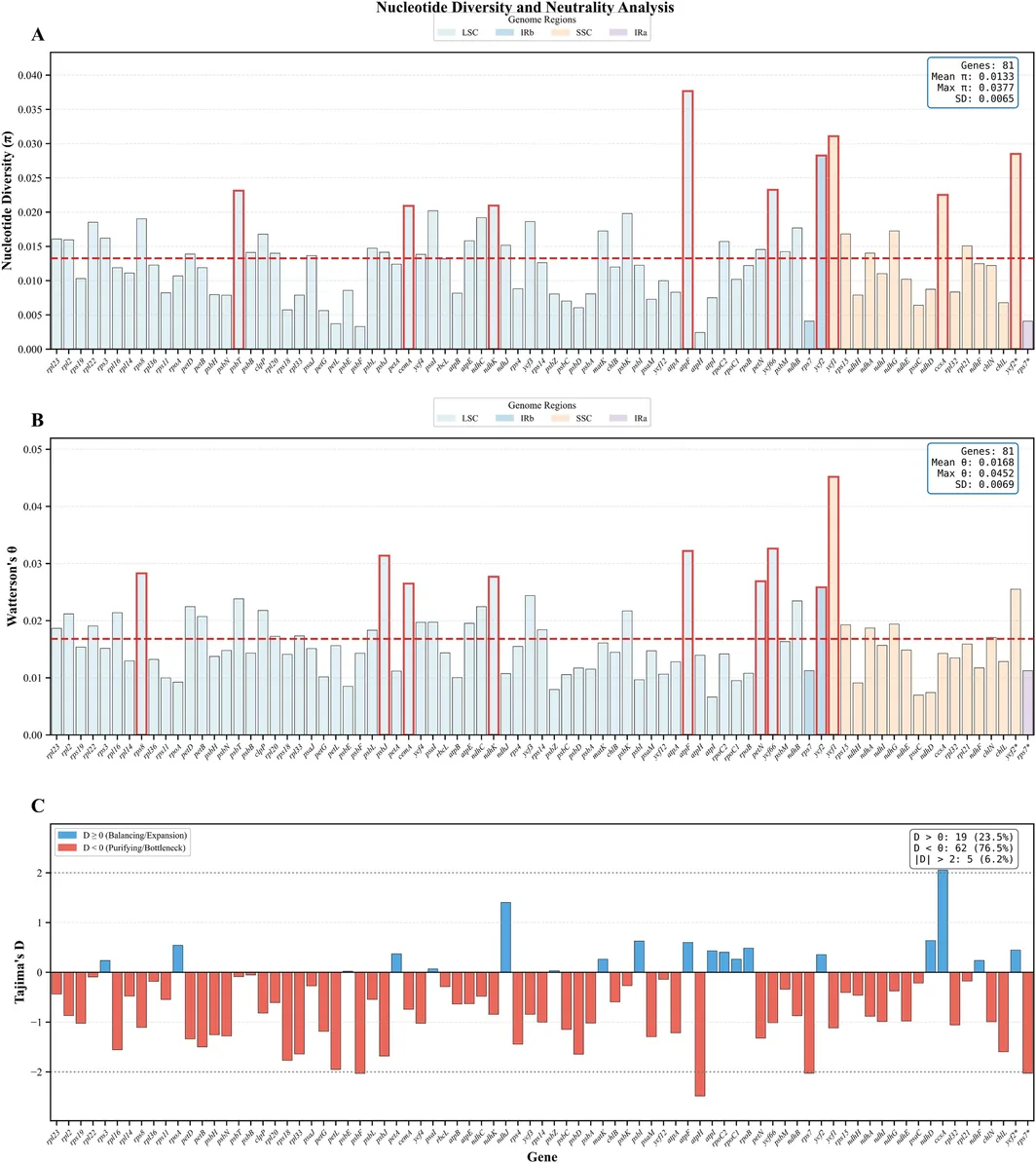
(A) Nucleotide diversity (π) across genes. The dashed horizontal line indicates the mean π value across all genes. (B) Watterson's estimator (θ) across genes. The dashed horizontal line indicates the mean θ value. (C) Tajima's *D* values across genes. Positive values are compatible with balancing selection or population contraction, whereas negative values are compatible with purifying selection or population expansion. These interpretations should be regarded as statistical signals rather than direct evidence of demographic or selective processes.

To assess the level of DNA polymorphism and the evolutionary pressures acting on specific genes, we calculated nucleotide diversity (π), Watterson's estimator (θ), and Tajima's *D* for 81 shared protein‐coding genes across 60 *Isoetes* species (Table S5). The overall nucleotide diversity was relatively low, with an average π value of 0.0133 and a standard deviation of 0.0065 (Figure 12A). However, substantial variation was observed among the genomic regions. The small single copy (SSC) region presented the highest degree of polymorphism, whereas the inverted repeat (IR) regions were highly conserved, with the lowest diversity values. This aligns with the structural stability typically observed in the IR regions of land plants.

Among the 81 genes analyzed, the π values ranged from near 0 to a maximum of 0.0377. We identified several hypervariable regions (divergence hotspots) that exceeded the average background variation. The gene *atpF* presented the highest nucleotide diversity (π = 0.0377), followed by *ycf1*, *ccsA, matK, ndhF,* and *rpl32*. Watterson's estimator showed a similar trend (Figure 12B), with a mean θ value of 0.0168 and a maximum of 0.0452, further confirming that *ycf1* and the *ndh* gene family represent the most rapidly evolving loci in the *Isoetes* chloroplast genome (Dong et al. 2015; Neubig et al. 2009).

Tajima's *D* analysis was conducted to screen for deviations from neutral expectations (Figure 12C). The results revealed that the majority of genes (62 genes, 76.5%) presented negative Tajima's *D* values, whereas only 19 genes (23.5%) presented positive values. Notably, 5 genes (6.2%) displayed significant deviations from neutrality, with absolute values greater than 2. The prevalence of negative Tajima's *D* values is compatible with an excess of low‐frequency variants, which may reflect purifying selection, demographic effects, or lineage‐level sampling structure (Tajima 1989; Carlson et al. 2005). Alternatively, this pattern could reflect recent population expansion following a bottleneck event. Genes with strongly negative Tajima's *D* values, such as *ndhF* and *ccsA*, may represent loci with unusual polymorphism patterns, but distinguishing among purifying selection, demographic effects, and lineage‐specific sampling requires additional population‐level data.

## 4 Discussion

### 4.1 Evolutionary Forces Shaping CUB in *Isoetes* Chloroplast Genomes

As one of the most ancient extant lineages of aquatic vascular plants, *Isoetes* occupies a unique phylogenetic position that provides an exceptional opportunity to investigate long‐term evolutionary processes acting on chloroplast genomes (Karol et al. 2010). The chloroplast genome, characterized by relative structural conservation and predominantly maternal inheritance, has long been regarded as a robust molecular system for reconstructing phylogenetic relationships, tracing species divergence, and inferring evolutionary mechanisms in plants (Pereira, Giulietti, Pires, et al. 2021). In *Isoetes*, this utility is further enhanced by the lineage's deep evolutionary history, ecological specialization, and persistence in extreme or fluctuating aquatic environments (Wickell et al. 2021). Together, these features make *Isoetes* an informative model for understanding how mutation pressure, natural selection, and functional constraints interact to shape CUB over extended evolutionary timescales (Hershberg and Petrov 2008).

Rather than treating the multiple analyzes as independent descriptive surveys, we interpreted them in relation to the three hypotheses proposed in the Background. Overall, the results support the first hypothesis that AT‐biased mutation pressure establishes the broad compositional background of *Isoetes* chloroplast codon usage, as indicated by low GC3s values, universal A/U‐ending preferred codons, and high ENC values. The second hypothesis was also partially supported: codon usage constraints were not evenly distributed across all genes but were concentrated mainly in photosystem and ribosomal protein genes, whereas Ka/Ks analyzes showed that purifying selection predominates at the protein‐coding level. The third hypothesis was supported by the coexistence of highly conserved plastome structure with localized variation in SSRs and several hypervariable loci. Together, these findings indicate that chloroplast genome evolution in *Isoetes* is best understood as a balance between genome‐wide mutational bias, gene‐specific functional constraints, and localized sequence divergence.

The combined codon usage analyzes provide complementary, rather than redundant, evidence for the balance between mutation pressure and selective constraints in *Isoetes* chloroplast genomes. Base composition, RSCU, and ENC values first establish the overall pattern: codon usage bias is relatively weak, but it is consistently structured by an AT‐rich background and a universal preference for A/U‐ending codons (Wright 1990). This pattern supports the hypothesis that AT‐biased mutation pressure plays a major role in shaping the genome‐wide compositional background.

The ENC‐GC3s analysis refines this interpretation by identifying where codon usage deviates from mutational expectation. Although many gene records follow the general expectation associated with GC3s composition, 22.91% showed marked deviation from Wright's expected curve. These deviations were concentrated mainly in photosystem and ribosomal protein genes, indicating that selective or translational constraints act most clearly on specific functional modules rather than uniformly across the entire chloroplast genome.

PR2 and neutrality plot analyzes further clarify the nature of this mutation–selection balance. PR2 analysis revealed asymmetric third‐position nucleotide usage, particularly a T/G bias, suggesting that synonymous sites are influenced by directional mutational or strand‐specific processes. In contrast, the weak relationship between GC3 and GC12 in the neutrality plots indicates that variation at synonymous third positions is not strongly transmitted to the first and second codon positions, which are more directly constrained by amino acid composition and protein function. Thus, mutation pressure appears to establish the AT‐rich synonymous background, whereas purifying selection and functional constraints restrict variation at more functionally sensitive codon positions.

COA provides an exploratory multivariate summary of these patterns. Because the first two axes explained only a limited proportion of total codon usage variation, the absence of strong clustering should not be overinterpreted as genome‐wide homogeneity. Instead, it is consistent with the view that major codon usage trends are broadly similar across *Isoetes* chloroplast genes, while more specific variation is distributed across functional categories and additional dimensions. Taken together, these analyzes support a model in which genome‐wide mutational bias, gene‐specific codon usage constraints, and protein‐level purifying selection jointly shape chloroplast codon evolution in *Isoetes* (Hershberg and Petrov 2008; Rocha 2004).

### 4.2 Functional Implications and Practical Significance of Codon Usage Patterns

Although CUB in *Isoetes* chloroplast genomes is modest overall, its potential functional implications warrant further investigation. In many organisms, codon usage patterns have been associated with translational efficiency, translational accuracy, and gene expression level (Quax et al. 2015). In the present study, the marked deviation of photosystem and ribosomal protein genes from the expected ENC curve is consistent with the possibility of stronger functional or translational constraints on these essential chloroplast genes. However, because transcript abundance, protein expression, and plastid tRNA availability were not analyzed, this pattern should not be interpreted as direct evidence of translational optimization. Instead, these genes should be regarded as candidates for future studies integrating codon usage, gene expression, and chloroplast translational dynamics.

The pronounced AT enrichment at synonymous sites may have functional implications beyond simple nucleotide composition, but its ecological relevance remains uncertain. The observed T/G bias at the third codon position could potentially be associated with lineage‐specific mutational processes or chloroplast genome maintenance, rather than necessarily reflecting direct adaptation to particular environmental factors. Although wetland habitats may expose *Isoetes* species to fluctuating light, redox, and water conditions (Xu et al. 2014), the present study does not directly test relationships between codon usage and environmental variables. Future studies integrating ecological metadata, transcriptomic profiles, and experimental stress‐response data will be necessary to evaluate whether these codon usage patterns have adaptive significance.

From an applied perspective, the elucidation of codon usage patterns in *Isoetes* chloroplast genomes has practical implications for chloroplast genetic engineering and conservation genomics. The identification of preferred codons may provide useful reference information for future studies of chloroplast gene expression or codon optimization, although practical applications in *Isoetes* would require substantial functional validation (Rocha 2004). Moreover, understanding the balance between conservation and flexibility in chloroplast genomes can inform predictions about the evolutionary resilience of *Isoetes* under ongoing environmental change.

It is important to distinguish statistical associations from biological causation when interpreting codon usage and SSR variation. The present analyzes reveal compositional patterns, distributional biases, and associations among genomic features, but they do not directly demonstrate the mechanistic causes of these patterns. For example, the enrichment of A/U‐ending codons is consistent with AT‐biased mutation pressure, and the concentration of markedly deviating ENC‐GC3s records in photosystem and ribosomal protein genes is compatible with gene‐specific functional or translational constraints. However, direct causal links between codon usage, translational efficiency, gene expression, or ecological performance cannot be established without transcriptomic, proteomic, tRNA abundance, or experimental validation. Similarly, the predominance of A/T‐rich SSRs and their preferential localization in noncoding regions are consistent with replication slippage and weaker functional constraints in noncoding regions, but these mechanisms should be regarded as plausible explanations rather than demonstrated causal processes. Therefore, we interpret CUB and SSR patterns as genomic associations that generate testable hypotheses for future functional and ecological studies.

### 4.3 SSR Dynamics and Evolutionary Constraints in *Isoetes* Chloroplast Genomes

SSRs are widely regarded as neutral or near‐neutral molecular markers that reflect underlying mutational processes and genome dynamics. In ancient plant lineages with low GC contents, such as *Isoetes*, SSR analysis is both theoretically informative and practically valuable for studying genome evolution, genetic diversity, and population structure (Wanga et al. 2021; Niu et al. 2023; Li et al. 2020). In the present study, we conducted a comprehensive survey of SSR distribution across the chloroplast genomes of 60 *Isoetes* species, revealing patterns consistent with strong mutational bias and structural constraints.

Mononucleotide repeats, particularly A/T motifs, dominate the SSR landscape of *Isoetes* chloroplast genomes, a pattern commonly observed in AT‐rich plant plastomes (Niu et al. 2023). This distribution is consistent with the AT‐rich nucleotide composition of the chloroplast genome and suggests that AT‐biased mutational processes may influence repeat composition (Provan et al. 2001). However, SSR abundance alone cannot establish the precise mutational mechanism responsible for repeat accumulation. Although replication slippage is a plausible explanation for the prevalence of short mono‐ and dinucleotide repeats, direct evidence would require population‐level polymorphism data or experimental assessment of repeat‐length variation (Ebert and Peakall 2009).

The preferential localization of SSRs in intergenic regions is also best interpreted as an association rather than direct proof of causal constraint. Noncoding regions may tolerate repeat expansion or contraction more readily than protein‐coding regions, whereas coding regions are expected to be more sensitive to indels that disrupt reading frames or alter protein structure. Thus, the observed SSR distribution is consistent with weaker functional constraints in noncoding regions and stronger constraints in coding regions. Nevertheless, the potential functional roles of conserved SSR motifs in chloroplast DNA replication, repair, or transcription remain hypothetical and require targeted experimental and comparative validation.

Despite the broad geographic distribution and ecological diversity of *Isoetes*, the chloroplast genome structure remains remarkably conserved across species (Karol et al. 2010). The genome size variation is minimal (142,880–145,535 bp), and the overall GC content is highly stable. Codon usage patterns and RSCU values are likewise highly consistent, indicating that *Isoetes* chloroplast genomes are subject to exceptionally strong functional constraints. This extreme conservation suggests that core chloroplast functions, particularly photosynthesis, impose stringent limits on structural and compositional divergence, leaving limited room for large‐scale structural or compositional divergence at the plastome level.

The SSR patterns observed in *Isoetes* provide insight into plastome evolution beyond the simple presence of AT‐rich repeats. The dominance of mono‐ and dinucleotide SSRs indicates that replication slippage may be a major source of fine‐scale sequence variation in these chloroplast genomes. However, the nonrandom distribution of SSRs among genomic regions suggests that their accumulation is filtered by functional constraints. SSRs were preferentially located in noncoding regions, where repeat length variation is less likely to disrupt gene function, whereas their reduced occurrence in coding regions implies purifying selection against indels that could cause frameshifts or alter conserved chloroplast proteins. Therefore, SSR distribution reflects an interaction between local mutational processes and selective constraints on plastome function.

Importantly, the coexistence of conserved and species‐specific SSR motifs provides a useful framework for conservation genomics. Conserved motifs may represent stable mutational hotspots shared across the genus, whereas species‐specific SSRs are likely to be informative for identifying closely related taxa and evaluating genetic variation within endangered populations. Given the threatened status of many *Isoetes* species, these SSR loci could be developed into chloroplast markers for phylogeographic reconstruction, population monitoring, and germplasm conservation.

### 4.4 Selection Patterns and Gene‐Level Evolutionary Dynamics

Ka/Ks analysis provides a powerful framework for disentangling the relative contributions of natural selection and mutation pressure to gene evolution (Wei et al. 2025). Our results show that the vast majority of chloroplast genes in *Isoetes* present Ka/Ks ratios well below 1, indicating pervasive purifying selection. This finding is consistent with the essential and conserved roles of chloroplast genes in photosynthesis, carbon fixation, and gene expression.

The elevated Ka/Ks ratios observed in several NDH complex genes, particularly ndhC, should be interpreted with caution. Although Ka/Ks values greater than 1 are often considered candidate indicators of positive selection, pairwise Ka/Ks comparisons alone cannot distinguish adaptive evolution from relaxed selective constraints. In chloroplast genomes, elevated Ka/Ks ratios may also arise from reduced functional constraints, lineage‐specific rate acceleration, low synonymous substitution rates, or structural changes affecting gene function. Therefore, the high Ka/Ks values detected for ndhC may indicate relaxed purifying selection, lineage‐specific rate variation, or possible functional divergence. This interpretation is consistent with the broader distribution of Ka/Ks values observed for the NDH complex compared with the strong conservation of photosystem and ribosomal protein genes. Future studies using branch‐site models, site‐specific selection tests, transcriptomic data, and functional validation will be necessary to determine whether the elevated Ka/Ks ratios in ndhC reflect true positive selection or relaxed functional constraint.

The exceptionally low Ka/Ks ratio observed for the *rbcL* gene highlights the extreme functional constraint acting on RuBisCO, a key enzyme in the Calvin cycle (Vitlin Gruber and Feiz 2018). Even minor alterations in this protein can have profound effects on photosynthetic efficiency, explaining the strong selective pressure against nonsynonymous substitutions. In contrast, elevated Ka/Ks values in certain genes, such as ndhC, suggest relaxed constraints or possible lineage‐specific divergence, although their relationship with environmental responsiveness of the NDH complex requires further testing (Martín and Sabater 2010). This heterogeneity underscores the fact that chloroplast genomes are not uniformly constrained but instead exhibit gene‐specific evolutionary trajectories (Zhang et al. 2022).

At the gene family level, carbon fixation‐related genes, RNA polymerase genes, and ribosomal protein genes display strongly left‐skewed Ka/Ks distributions, reinforcing their fundamental importance in maintaining cellular homeostasis. Conversely, the broader and more dispersed Ka/Ks distributions observed in NDH dehydrogenase‐related genes may reflect relaxed constraints, functional divergence, or lineage‐specific rate variation, although links to environmental factors require further testing. These patterns suggest that, while the overall chloroplast genome is highly conserved, certain functional modules show gene‐specific evolutionary flexibility; however, their ecological relevance requires further testing.

### 4.5 Genome Structure Conservation and Evolutionary Divergence

Phylogenetic and collinearity analyzes revealed that *Isoetes* follows a distinctive evolutionary trajectory characterized by moderate radiation and the formation of intermediate‐sized lineage branches. Geographic isolation and habitat heterogeneity may be associated with lineage differentiation, but their direct influence on codon usage and gene evolution remains to be tested. Nevertheless, closely related species exhibit high collinearity and nearly identical gene arrangements, underscoring the tight structural constraints acting on chloroplast genomes.

Although the phylogeny was reconstructed using IQ‐TREE with model selection and dual branch‐support assessment, it should still be regarded primarily as a comparative framework rather than a definitive species‐level phylogeny because several nodes within the Isoetes clade remained weakly supported and chloroplast protein sequences provided limited resolution among closely related species. Nevertheless, the major conclusions of this study are primarily based on codon usage indices, ENC‐GC3s deviation analysis, SSR distribution, Ka/Ks patterns, nucleotide diversity, and plastome collinearity, and therefore do not depend solely on fine‐scale phylogenetic resolution.

Compared with its close relative *Selaginella*, *Isoetes* displays a more conservative codon usage pattern and a stronger AT bias (Mower and Vickrey 2018). The remarkable stability of the IR region further contributes to overall genome conservation, while subtle IR boundary variations may serve as indicators of evolutionary divergence. These differences likely reflect contrasting ecological strategies: as a relict lineage, *Isoetes* shows a highly conservative plastome architecture, which may be associated with its ancient lineage history and long‐term functional constraints.

The long‐term conservation of gene order, orientation, and functional regions across *Isoetes* species suggests that chloroplast genome stability is a key component of the evolutionary success of this genus. This stability may be linked to its ancient origin and niche specialization (Yang, Yu, et al. 2022), consistent with long‐term functional conservation rather than rapid structural innovation.

It should be emphasized that the present study is based primarily on comparative chloroplast genomic analyzes. Therefore, interpretations regarding ecological adaptation or functional consequences of codon usage remain inferential. Direct evidence will require integration of ecological variables, gene expression profiles, plastid translational data, and experimental validation under controlled environmental conditions.

### 4.6 Ecological Relevance and Limitations of Environmental Inference

Although *Isoetes* species occupy diverse aquatic and semiaquatic habitats, the present study did not incorporate external ecological or environmental datasets, such as occurrence‐based climatic variables, hydrological parameters, nutrient status, or habitat stability. Therefore, the observed genomic patterns should not be interpreted as direct evidence of ecological adaptation. Instead, they identify candidate genomic features that may be useful for future ecological‐genomic studies. For example, variation in GC3s, ENC, ΔENC values, SSR density, nucleotide diversity, and Ka/Ks ratios in particular gene families could be tested against environmental variables such as temperature seasonality, precipitation, elevation, hydroperiod, light availability, and water chemistry. Such analyzes would allow explicit evaluation of whether codon usage patterns, repeat dynamics, or gene‐level evolutionary rates are associated with habitat heterogeneity or environmental stress.

The lack of comparable ecological metadata across all 60 *Isoetes* species currently limits formal genotype–environment association analyzes. Many *Isoetes* taxa are rare, geographically restricted, or represented by limited occurrence records, which may introduce sampling bias when extracting environmental variables from public databases. For this reason, we interpret the ecological relevance of the observed CUB and plastome patterns cautiously. Future studies integrating high‐quality occurrence data, standardized habitat measurements, ecological niche modeling, and transcriptomic or proteomic profiles will be necessary to test whether the genomic features identified here are associated with environmental adaptation or ecological differentiation in *Isoetes*.

## 5 Conclusion

This study provides a genus‐wide comparative analysis of chloroplast codon usage and plastome evolution across 60 *Isoetes* species. The results show that *Isoetes* chloroplast genomes are highly conserved in genome size, GC content, IR structure, and gene organization. Codon usage bias is generally weak but consistently structured, with all preferred codons ending in A or U and GC3s values remaining low across species.

Multiple lines of evidence indicate that AT‐biased mutation pressure is the dominant factor establishing the overall codon usage background, whereas gene‐specific constraints further shape particular functional categories. Quantitative ENC‐GC3s analysis showed that markedly deviating gene records were concentrated mainly in photosystem and ribosomal protein genes, suggesting stronger codon usage constraints in essential chloroplast functional modules. Ka/Ks analysis indicated that purifying selection predominates across most chloroplast genes, while elevated Ka/Ks ratios in some NDH genes, especially ndhC, may reflect relaxed selective constraints or lineage‐specific divergence rather than definitive positive selection.

The SSR loci, hypervariable regions, and codon usage features identified here provide useful genomic resources for phylogenetic and conservation studies of Isoetes. However, ecological or functional interpretations of these patterns remain inferential. Future studies integrating environmental variables, transcriptomic data, plastid translational profiles, and experimental validation will be necessary to test whether the genomic patterns identified here are associated with ecological differentiation or adaptive significance.

## Author Contributions

**Boxiong Li:** data curation (equal), formal analysis (equal), methodology (equal), validation (equal), writing – original draft (equal), writing – review and editing (equal). **Ying Xue:** writing – original draft (equal), writing – review and editing (equal). **Dingqi Zhang:** writing – original draft (equal), writing – review and editing (equal). **Guy Smagghe:** writing – review and editing (equal). **Shixiong Liang:** writing – original draft (equal), writing – review and editing (equal). **Shujing Wang:** writing – original draft (equal), writing – review and editing (equal). **Yuye Ge:** writing – original draft (equal), writing – review and editing (equal). **Zubaidai Aimaitijiang:** writing – original draft (equal), writing – review and editing (equal). **Yunpeng Gai:** conceptualization (lead), data curation (equal), formal analysis (equal), funding acquisition (lead), methodology (equal), project administration (lead), software (lead), validation (equal), writing – original draft (equal), writing – review and editing (equal).

## Funding

This research was funded by the General Program of the Beijing Association of Higher Education (Grant MS2024091) and the National Undergraduate Training Program on Innovation and Entrepreneurship (Grants 202510022481 and 202510022484).

## Ethics Statement

The authors have nothing to report.

## Consent

The authors have nothing to report.

## Conflicts of Interest

The authors declare no conflicts of interest.

## Supporting information


**Table S1:** Gene‐level genomic characteristics, nucleotide composition, and codon usage metrics for the complete chloroplast genomes of 60 Isoetes species. Each worksheet represents one Isoetes species and includes the GenBank accession number, gene annotation, gene type and length, nucleotide composition, GC content and GC skew, codon‐position‐specific GC contents, GC3s, and codon usage indices for protein‐coding genes, including relative synonymous codon usage (RSCU), effective number of codons (ENC), and codon adaptation index (CAI).

**Table S2:** Characteristics and genomic distribution of simple sequence repeats (SSRs) identified in the chloroplast genomes of 60 Isoetes species. For each SSR locus, the table provides the repeat type, motif length, genomic start and end positions, repeat‐unit sequence, repeat number, associated gene(s), and genomic location category (coding sequence, intron, or intergenic spacer).

**Table S3:** ENC–GC3s deviation analysis of chloroplast protein‐coding genes across 60 Isoetes species. The table includes observed effective number of codons (ENC), GC3s, expected ENC values under Wright's neutral expectation, and the deviation between observed and expected ENC (ΔENC = ENCobs − ENCexp) for 4523 gene records, together with summaries by functional category, gene, and species. Records with ΔENC < 0 were classified as lying below the expected ENC curve, whereas records with ΔENC < −5 were defined as markedly deviating records.

**Table S4:** Nonsynonymous and synonymous substitution rates and Ka/Ks ratios for orthologous chloroplast protein‐coding gene comparisons among Isoetes species. The table provides Ka, Ks, and Ka/Ks values together with gene identities and functional categories, as well as gene‐ and gene‐family‐level summary statistics used to characterize variation in selective constraints among chloroplast genes.

**Table S5:** Nucleotide diversity and related evolutionary statistics for 81 shared chloroplast protein‐coding genes across 60 Isoetes species. The table summarizes nucleotide diversity (π), Watterson's estimator (θ), Tajima's *D*, and haplotype diversity (Hd), together with gene location, sequence composition, and substitution‐rate statistics used for comparative analyzes of chloroplast gene diversity and evolutionary variation.

## Data Availability

All the data generated or analyzed during this study are included in this published article and its Supporting Information files.
